# The global biomass and number of terrestrial arthropods

**DOI:** 10.1126/sciadv.abq4049

**Published:** 2023-02-03

**Authors:** Yuval Rosenberg, Yinon M. Bar-On, Amir Fromm, Meital Ostikar, Aviv Shoshany, Omer Giz, Ron Milo

**Affiliations:** Department of Plant and Environmental Sciences, Weizmann Institute of Science, Rehovot, Israel.

## Abstract

Insects and other arthropods are central to terrestrial ecosystems. However, data are lacking regarding their global population abundance. We synthesized thousands of evaluations from around 500 sites worldwide, estimating the absolute biomass and abundance of terrestrial arthropods across different taxa and habitats. We found that there are ≈1 × 10^19^ (twofold uncertainty range) soil arthropods on Earth, ≈95% of which are soil mites and springtails. The soil contains ≈200 (twofold uncertainty range) million metric tons (Mt) of dry biomass. Termites contribute ≈40% of the soil biomass, much more than ants at ≈10%. Our estimate for the global biomass of above-ground arthropods is more uncertain, highlighting a knowledge gap that future research should aim to close. We estimate the combined dry biomass of all terrestrial arthropods at ≈300 Mt (uncertainty range, 100 to 500), similar to the mass of humanity and its livestock. These estimates enhance the quantitative understanding of arthropods in terrestrial ecosystems and provide an initial holistic benchmark on their decline.

## Teaser

Synthesizing local data quantifies arthropods in terrestrial ecosystems and provides an initial benchmark on their decline.

## INTRODUCTION

With Earth entering the age of the Anthropocene, human domination of the planet affects myriad ecological and geological processes. The elevated rates of current species extinctions are estimated to be orders of magnitude above the background rates (i.e., the rate of extinction before humans became a primary contributor to extinctions) (*1*, *2*). Many extant vertebrate species, such as amphibians (*3*) and birds (*4*), are suffering from rapidly decreasing populations, even if they are not currently at risk of extinction (*5*).

Recent studies (*6*–*12*) have likewise observed marked declines in the populations of insects and other terrestrial arthropods, such as spiders, mites, and centipedes. Terrestrial arthropods are a major component of many food webs, and they engineer ecosystems and provide ecosystem services (*8*, *13*), resulting in a pressing need to better understand their global abundance and dynamics. Most land arthropod population studies are based on sampling techniques that can measure trends in population or biomass but do not measure their absolute inventory over an area. While these measures are informative for population decline, knowing the absolute quantities of arthropods in different habitats and locations can help us integrate the observed trends, place them in a wide perspective, and better understand the wide ecological significance of terrestrial arthropods. For example, the knowledge of the global biomass distribution of termites allows estimating their contribution to global warming through the greenhouse gases they produce (*14*). Likewise, standing biomass estimates of arthropods may improve our understanding of food webs and of possible effects of declines in arthropod numbers [e.g., (*13*, *15*)].

Current knowledge of the global distribution of terrestrial arthropods is lacking. Soil fauna across biomes was investigated in the 1960s and 1970s (*16*), with newer studies refining our knowledge for key groups (*17*–*19*). We have recently estimated the global biomass of terrestrial arthropods (*20*). Nevertheless, our estimates were coarse and covered limited data. Here, we refine and substantiate the global estimates by aggregating and analyzing much more data. Specifically, we extended the number of samples used to infer the biomass of soil arthropods by more than an order of magnitude relative to previous efforts (*16*, *19*, *20*). We included human-dominated landscapes (croplands and pastures) and incorporated data on groups for which global estimates were lacking. We also analyzed area-based data for above-ground arthropods, but we note that these data are especially scarce. We estimate the global biomass and population size of terrestrial arthropods and provide a holistic view of their taxonomic composition and distribution. This collection reveals the current state of quantitative knowledge on the global abundance of arthropods and highlights key knowledge gaps, which future research should help close.

## RESULTS

**Fig. 1.**
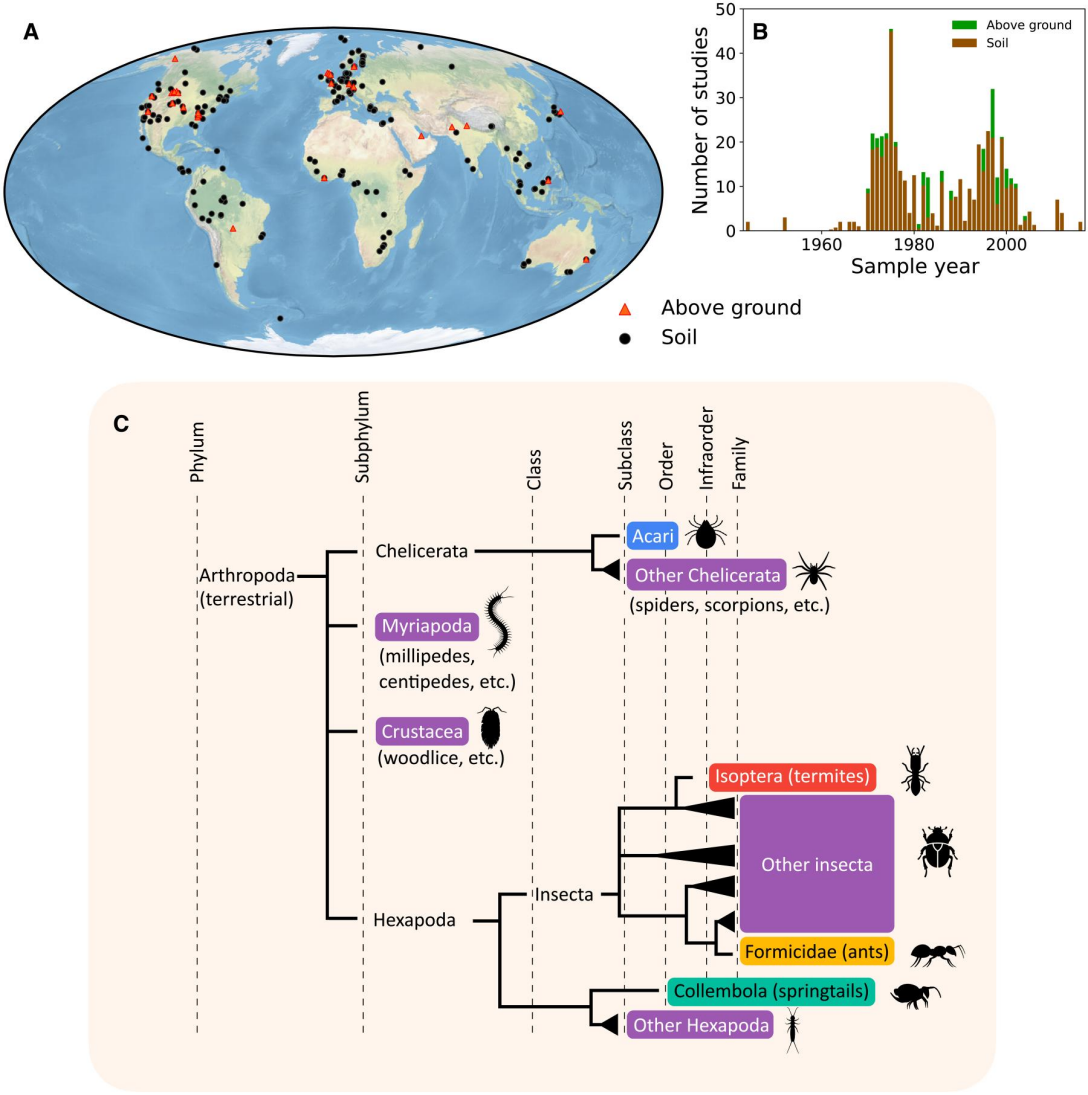
Locations, years, and taxonomic grouping of the arthropod abundance measurements used in this study. (**A**) A global map of the sampling locations in our dataset (see text for habitat type definitions). (**B**) Sampling year distribution of the sites in (A) (see the Supplementary Materials for further details). (**C**) A phylogenetic tree of taxonomic groups for our analysis of soil arthropods. Each color represents a single aggregated taxonomic group. Above-ground arthropods were analyzed separately as a single taxonomic group.

We scoured the literature for measurements of absolute population and biomass densities of arthropods and collected ≈7000 such measurement-based evaluations across ≈500 sites in ≈300 different locations (one location can have several sampling sites, see Methods) over many years, as shown in Fig. 1 (A and B). The studies cover all major biomes (see fig. S3 for the biome definitions). Overall, we obtained data for ≈390 sites with measured biomass density (measured in units of mass per area) and ≈330 sites with measured population densities (measured in number of individuals per area), with ≈200 of them estimating both biomass and population.

We integrated the data for global extrapolation by first dividing it based on habitat type, biome, and taxonomy (see Methods). We focused on two distinct habitat types: soil and plant litter and above-ground (i.e., surface-dwelling and plant-associated arthropods). The main reason for separating these habitats is that the techniques for sampling arthropod communities in them are quite different [e.g., core sampling for soils and trap removal, suction sampling, or canopy fogging (insecticide knockdown) for above-ground communities]. Thus, they are often studied separately and also differ in the quality and breadth of available data. Soil arthropods were sampled in ≈440 sites, and above-ground arthropods were sampled in ≈60 sites.

For the soil and plant litter habitats, portions of soil and litter were typically directly sampled under random quadrat counts, followed by a variety of extraction methods such as hand sorting and extraction funnels. These techniques collect a wide community assemblage of soil arthropods and estimate their local absolute inventory. However, ants and termites are key taxonomic groups that often require more specific techniques to properly estimate their absolute inventory. This stems from their population being concentrated in sparsely distributed colonies that are harder to sample (*21*). To compare and integrate the observed soil arthropods’ densities, we divided them on the basis of their taxonomy and available data into five groups: Acari (mostly mites), Collembola (springtails), Blattodea: Isoptera (termites), Hymenoptera: Formicidae (ants), and all other arthropods (which we call “others”), as depicted in Fig. 1C.

For the above-ground habitats, we used samples of wide taxonomic compositions and analyzed the above-ground arthropods as a single taxonomic group. We relied on sampling techniques that were meant to sample the entire above-ground arthropod community (such as canopy fogging or trap removal). Nevertheless, these techniques are also likely to undersample parts of the arthropod community. Taxonomic groups that are likely to be underrepresented, while possibly contributing a substantial share of the global biomass (e.g., lepidopterans), were analyzed separately (see Supplementary Materials).

**Fig. 2.**
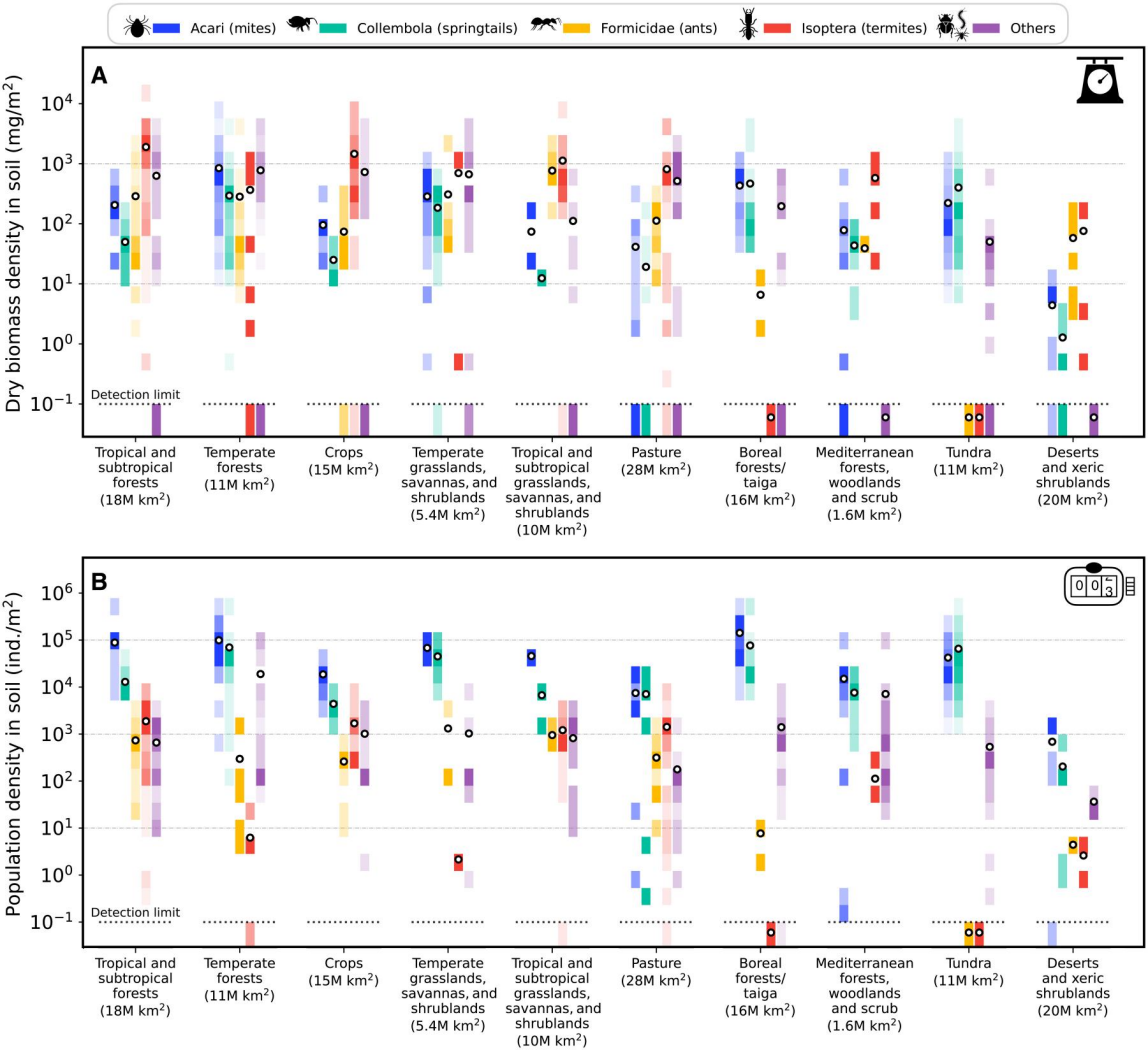
Biomass and population densities of soil arthropod groups across global biomes. In each biome and for each group of arthropods, biomass densities (**A**) or population densities (**B**) reported in the different sites are binned evenly in log scale. Each bin extends to roughly twice its minimal value. The shading of each bin represents the fraction of datapoints lying in the bin, with the darkest color bin having the highest number of measurements for a specific taxonomic group and biome. Black circles mark the mean density. The dashed lines represent the detection limit, the lowest available nonzero measurement. The biomes are in descending order with respect to their total arthropod biomass density. The area of each biome is given in parentheses in units of millions of square kilometers (1M km^2^ = 10^6^ km^2^ = 10^12^ m^2^). See Methods for the definitions of biomes.

Figure 2 shows the distribution of average biomass density (Fig. 2A) and average population density (Fig. 2B) of soil arthropods across sites for each global terrestrial biome. Summary statistics are also found in tables S3 and S4. We find that the density distributions range over several orders of magnitude. They typically resemble a log-normal distribution for taxa that have many datapoints in a biome and have a more scattered distribution for taxa with fewer datapoints. We find that the average dry biomass density of all soil arthropods ranges from roughly 3 g/m^2^ in tropical and subtropical forests (dominated by termites) down to about 0.1 g/m^2^ in deserts and xeric shrublands. The population density is dominated by mites and springtails and ranges from about 200,000 individuals/m^2^ in boreal forests down to roughly 1000 individuals/m^2^ in deserts and xeric shrublands.

**Fig. 3.**
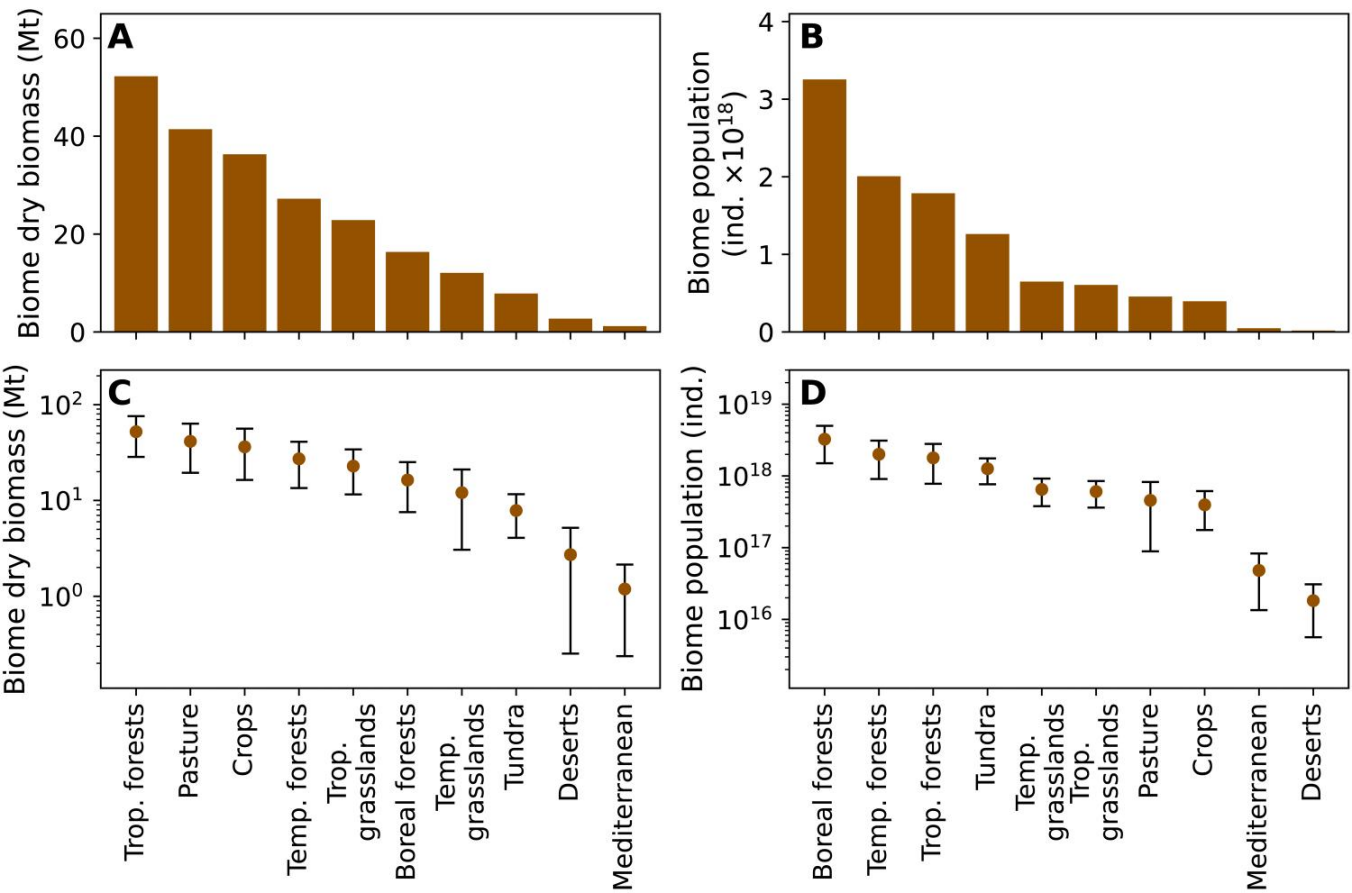
Total biomass and number of individuals of soil arthropod across biomes. For each biome, panels (**A**) and (**C**) show the total dry biomass on linear and logarithmic scales, respectively. Panels (**B**) and (**D**) show the same for total populations. Error bars represent 95% confidence intervals. Biomes are as in Fig. 2, with shortened names.

**Fig. 4.**
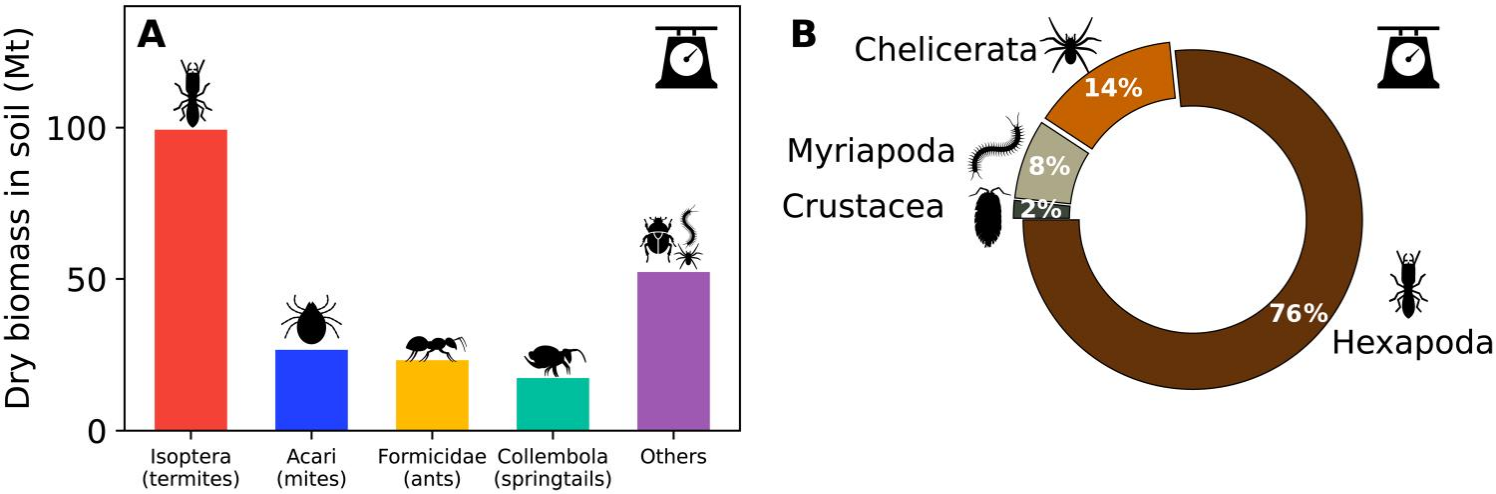
Taxonomic distribution of the total biomass of soil arthropods. (**A**) Biomass distribution between the different taxonomic groups of soil arthropods, as defined in the main text. (**B**) Comparison between the biomass of the different subphyla of soil arthropods.

We find the total biomass and population of soil arthropods in each biome (Fig. 3) by multiplying average densities by the global area of the biome using a bootstrapping procedure. We estimate the total mass of soil arthropods to be ≈200 (uncertainty range, 100 to 400) million metric tons (Mt) of dry weight (see Methods for further details). Termites contribute about 40% of the biomass in the soil; ants, springtails, and mites contribute about 10% each; and other soil arthropods contribute the remainder, as shown in Fig. 4A. We estimate the total population of soil arthropods to be ≈1 × 10^19^ (uncertainty range, 0.5 × 10^19^ to 2 × 10^19^) individuals. The microarthropods, mites and springtails, account for >95% of the total population of soil arthropods, with about two-thirds of these being mites.

We find that above-ground arthropods have a total of ≈50 Mt (uncertainty range, 20 to 100) of dry biomass, with our upper range potentially reaching ≈200 Mt when considering key biases (see Supplementary Materials). Together, both habitats contain a global terrestrial arthropod biomass of ≈300 Mt (uncertainty range, 100 to 500). The arthropods that are typically found above ground are larger than mites and springtails, which are mainly found in soils (*22*). Thus, population densities of above-ground arthropods are typically much smaller than those of soil arthropods and contribute little to the global number of individual arthropods.

Figure 4B compares the biomasses of soil arthropods with equivalent taxonomic ranks, aggregated by subphylum. Hexapoda (mainly insects and springtails) contribute the most to the total soil arthropod biomass (≈77%), followed by Chelicerata (e.g., spiders and mites) and Myriapoda (e.g., millipedes and centipedes), which contribute about 10% each. Crustacea (e.g., woodlice) account for only about 2%. Insects dominate the subphylum Hexapoda, with springtails accounting for only about 10% of its biomass in soils. We estimate that ants and termites alone constitute ≈55% of the total biomass of soil arthropods. Insects likely dominate the above-ground habitats as well, but the scarcity of data in these habitats does not allow us to robustly resolve their contribution.

## DISCUSSION

**Fig. 5.**
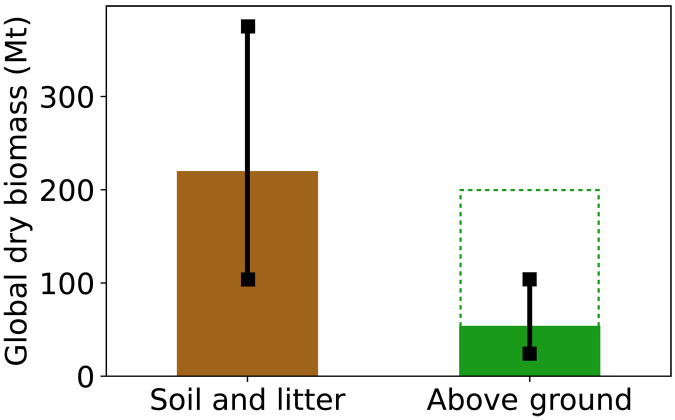
The global biomass of terrestrial arthropods. The total dry biomass in the Soil and plant litter habitat and in the Above-ground habitat. Error bars mark the uncertainty range calculated as the sum of the lower or upper bounds of all biome-level 95% confidence intervals. Dashed rectangle marks our supplementary upper estimate for the global biomass of above-ground arthropods.

We synthesized global arthropod abundance and biomass data from thousands of evaluations across ≈500 sampling sites, representing our current knowledge for most of the planet’s terrestrial biomes. This dataset reveals the global distribution of the biomass and populations of terrestrial arthropods, extending earlier work on these characteristic biomass densities (*16*, *19*). We estimate the total biomass of terrestrial arthropods at 300 Mt (uncertainty range, 100 to 500) of dry weight (Fig. 5) but discuss the existence of substantial caveats associated with this value below. Dry weight of 300 Mt is roughly equivalent to 1000 Mt of fresh weight (*16*) or 150 Mt of carbon (*20*). This improves upon our recent estimate of about 400 Mt of dry weight (*20*), which had a ≈15-fold uncertainty for the 95% confidence interval. Similarly, we estimate the total number of terrestrial arthropods at about 1 × 10^19^ individuals (uncertainty range, 0.5 × 10^19^ to 2 × 10^19^), which agrees with the range of 10^17^ to 10^19^ individuals estimated by Williams in the 1960s, based on limited data from U.K. soils (*23*).

While our estimates for the global biomass of soil arthropods are based on a reasonable amount of data, our estimates of the above-ground arthropod biomass are highly uncertain due to especially scarce data and possible biases in the sampling techniques, as discussed below. Thus, we first focus on characterizing the main trends found when analyzing the soil arthropod biomass and discuss the above-ground arthropod community separately.

When averaged globally, the biomass of soil arthropods is dominated by the subphylum Hexapoda, with termites representing ≈40% of the total mass (Fig. 4). We estimate that insects constitute about two-thirds of the total biomass of all soil terrestrial arthropods. Every square meter of top-soil contains tens of thousands of arthropods on average, the vast majority of which are tiny mites and springtails. All other arthropods combined have average densities of, at most, a few thousand individuals/m^2^. These findings generally agree with previous work on soil macroinvertebrates (*17*), where macroinvertebrate population densities were found to vary from ≈2000 individuals/m^2^ in tropical forests and savannas down to ≈200 individuals/m^2^ in temperate dry forests and shrubs. We also find general agreement between our biomass estimate for the group others and the corresponding data in (*17*) after converting it to biomass (see Supplementary Materials). Our estimated global biomass of termites (100 Mt; range, 50 to 170 Mt) agrees with previous estimates of 100 to 120 Mt (*14*, *24*) as well (*17*).

Our total population estimate for soil springtails (3 × 10^18^ individuals; range, 1 × 10^18^ to 6 × 10^18^ individuals) agrees with the recent estimate of 2 × 10^18^ individuals made in (*25*), but our total biomass estimate is about four times lower. This difference seems to originate from the large values of average body mass reported in (*25*), which are based on taxonomy and allometric evaluations. Studies in our dataset, such as (*16*), find much lower values by directly measuring the biomass of entire communities.

Our estimated global population of nonarboreal ants, considered as part of the soil and plant litter habitat, is 5 × 10^16^ (uncertainty range, 1 × 10^16^ to 9 × 10^16^) individuals, which agrees with the estimated range of 1 × 10^16^ to 6 × 10^16^ individuals in (*18*). The estimates in (*18*) are based on epigeic ants extracted from leaf litter, multiplied by a general correction factor to relate populations of captured workers to those of total colonies. Our separate estimate for soil ants’ dry biomass is ≈25 Mt (uncertainty range, 10 to 50 Mt) compared to 5 to 74 Mt estimated in (*18*). We note that (*18*) converted abundance to biomass estimates using an average of individual body mass calculated from a list of ant species body masses, without taking into account the relative abundance of each species in nature. This conversion can be improved by evaluating the weighted average body mass from whole communities, where their entire biomass and abundance are known. Our dataset yields a global average dry weight of 0.84 mg per soil ant. Using this value to convert the soil population range in (*18*) to biomass results in the same biomass range as the one reported by us. We also note that the estimate in (*18*) for arboreal ants is about a third of our estimate for the arthropod biomass in trees, in agreement with previous rough estimates for the fraction of ant biomass out of the total arboreal arthropod community (*24*).

The results we present here should be considered a tentative estimate due to the limited data available, which may be systematically biased in various ways. First, despite the remarkable increase in available data from previous estimates, it is still very limited, far from covering the highly variable distribution of arthropods in space and time. Second, the source data may be systematically biased in several ways, beyond the projected range due to random errors. Arthropod populations vary greatly with environmental changes (*10*, *11*), and sampling times are biased toward seasons of high abundance. Sampling locations (Fig. 1A) are biased toward rich countries, and some biomes are poorly covered. Substantial biases may also originate from the source studies’ design; methods of sampling, extraction, storage, and biomass evaluation; and publication process (*16*, *26*–*29*).

Our extrapolation methodology may have introduced additional biases. The aggregation of measurements based on biomes leaves out many important factors that influence the abundance of species. The distribution of arthropods varies between different locations within a biome due to many biogeographical factors (*30*), such as the physical environment (e.g., soil characteristics, altitude, and climate) and the local biotic environment (e.g., coevolving local plant and predator communities). Further dividing our limited data across relevant factors, however, introduces a statistical uncertainty in line with the bias-variance tradeoff (*31*).

To help assess the extent of these biases and their implications to our results, we conducted several sensitivity analyses. Sensitivity checks for the entire estimate of soil arthropods, such as dividing the biomes according to their biogeographical realms, resulted in about a 10% change in the overall biomass estimate (see Supplementary Materials). We also chose to focus on termites as the most dominant group of soil arthropods in terms of biomass (Fig. 4). Termites have substantial differences in abundance between continents (*32*, *33*), which implies that relying solely on biomes for spatial aggregation may result in a biased estimate of their biomass. Analyzing their biomass in a dedicated methodology (see Supplementary Materials), paying more attention to their unique biogeographical distribution, reduced their total biomass estimate by 20%. These sensitivity analyses indicate that our results are robust at the global level within the reported uncertainty ranges. Our biome-level estimates are more sensitive to these biases and should be considered with greater caution.

Most of the studies included in our dataset sampled only soil arthropods, and our estimates are less certain for other habitat types. Absolute biomass data for arboreal arthropods span all the tree-dominated biomes, but the number of sites sampled in each biome is especially limited. For tropical forests, which hold the highest biomass densities, we found only two studies that measure entire arboreal communities and report biomass per area of forest floor (see Supplementary Materials). This limits our ability to determine the natural variability in the local densities of above-ground arthropods. In addition, the sampling techniques used have varying efficiencies for different taxa and may undersample parts of the community. Caterpillars and adult lepidopterans, for example, are recognized as important constituents of the above-ground arthropod community (*34*) but may be underrepresented in studies using canopy fogging. For example, caterpillars may be affected by the insecticide fog but get locked into place by their crochets and thus not fall into the collecting trays.

We address these challenges by performing several supplementary analyses to establish an upper range for the total biomass of above-ground arthropods (see Supplementary Materials). First, we account for the low coverage of the natural variability of biomass densities within each biome using the local variability reported within sampled sites. For each biome, we calculate the 97.5th percentile of the biomass density per site. We then use the maximal value from these calculated densities as an upper range for the average biomass density in the biome. This results in an upper range of ≈150 Mt globally. Second, we account for possible undersampled taxonomic groups (such as lepidopterans and orthopterans) by generating crude upper estimates for their global biomass. Together, we can reach an upper estimate of ≈200 Mt of dry biomass for above-ground arthropods. This estimate is equivalent to extrapolating the highest observed biomass density for above-ground arthropods, excluding population outbreaks, over the entire tropical, subtropical, and temperate regions [density of 2.4 g/m^2^; (*35*)]. We note, however, that there is a possibility for underestimation in the value of ≈200 Mt despite the wide margins used.

Our biomass estimates are also consistent with the trophic interactions of terrestrial arthropods. We estimate the global yearly secondary productivity of herbivorous arthropods (including groups such as caterpillars and grasshoppers) to be ≈300 Mt, which agrees with a global average stock of less than 100 Mt (see Supplementary Materials). The global total annual arthropod prey is estimated at ≈500 Mt of dry biomass (see Supplementary Materials), including herbivorous arthropods as a major component. This is in line with our estimate of the herbivorous arthropods' secondary productivity.

Keeping the above caveats in mind, our synthesis provides a data-driven estimate toward the current global number and biomass of terrestrial arthropods. Our integrated dataset allows us to estimate global distributions of biomass across principal higher taxa and biomes and place their global biomass contributions in context. For example, we estimate that terrestrial arthropods have an order of magnitude higher biomass than wild mammals (*20*). Their biomass is similar to all humans and their livestock and also similar to the biomass of earthworms, enchytraeids, and nematodes combined (*20*). However, the biomass of terrestrial arthropods is an order of magnitude lower than marine arthropods (dominated by crustaceans) and two orders of magnitude lower than soil microbes (*20*). In contrast to mammals (*20*), the vast majority of terrestrial arthropods are still wild. Major domesticated species such as honey bees have a dry mass of only about 0.06 Mt [assuming a global stock of ≈100 million beehives (*36*), each containing ≈30,000 individuals (*37*, *38*) with ≈20 mg of dry body mass (*39*)]. Our data reveal the dominance of detritivores among arthropods and support the view of them as ecosystem engineers (*17*, *40*) that contribute considerably to plant decomposition (*41*) despite having much less biomass than plants, fungi, or bacteria (*20*). Our estimates show that soil and tropical insects should be given greater emphasis in assessments of global insect abundances decline [e.g., (*9*)]. Our findings could serve as a basis for future research on the ecological roles played by terrestrial arthropods, such as in carbon turnover and nutrient cycling (*16*).

Our global assessment highlights gaps in our current knowledge. We found limited data on flying insects, a guild for which reports suggest marked recent population losses (*6*, *8*, *9*), highlighting the need for better quantification of their global abundance. Substantial knowledge gaps also include the abundance of arthropods in deserts, soil microarthropods in croplands, and termites in general.

Ultimately, by building a knowledge base with an increased spatial, temporal, and taxonomic resolution, we could understand the basic environmental parameters that govern the global abundance of arthropods, as has been achieved for soil nematodes (*42*). Such a knowledge base will serve as a benchmark for monitoring the impact of humanity on arthropod abundance and the ecosystem functions they provide and for protecting their immense biodiversity.

## METHODS

### Study design

To estimate the global biomass and population of terrestrial arthropods, we collected relevant abundance data from published scientific articles, as detailed below, and analyzed it. We focused on data that can be extrapolated globally, such as biomass density of the entire arthropod assemblage, rather than measures of biomass intensity, or data specific for only certain species.

### Data collection

To collect as many samples of arthropod biomass and abundance, we have started our search with meta-analyses surveying the population and biomass densities of terrestrial arthropods. We have expanded on these initial studies by searching for broad terms such as “arthropods + biomass” in Google Scholar and specific search terms related to sampling techniques, biomes, and key taxonomic groups that require specific sampling, such as “Tullgren funnel,” “desert,” and “termites.” We have also used citation and author snowballing to extend the breadth of our dataset (*43*). For each sample, we have extracted the site at which the measurement was taken, as well as metadata such as the site location, biome, the type of habitat that was sampled (soil or above the ground), the taxon sampled, the sampling time (year and season), and the reported units for the sample (e.g., dry/wet weight). We kept track of the specific origin of the data in each study to allow us to trace back the source data. Overall, our dataset contains ≈100 studies, many of which are meta-analyses themselves. The data in our collection contain measurements of terrestrial arthropod biomass and population densities throughout the last century. We integrate data from several decades that represents, to a first approximation, the current state of the biomass and population size of arthropods.

### Data preprocessing

We filtered out inadequate data and standardized the units and metadata used before integrating the data and creating global estimates. In the filtering stage, we removed datapoints that did not measure the natural abundance of arthropods, such as samples that were artificially treated. In addition, we removed data from studies that reported the biomass or population of arthropods in units that are not useful for extrapolating densities of arthropods to larger areas (e.g., units reported per mass of foliage or per trap). We also removed studies that measured only a fraction of the arthropod population, as this was incompatible with the rest of our data and with our analysis procedure. The removed studies include those that measured only specific size fractions of the arthropod community or specific trophic modes such as herbivores.

In the harmonization stage, we converted all the data reported in the original studies into standard units that were consistent across all samples. These include conversion of biomass measurements into units of dry biomass density (in milligrams per square meter), which dominate the reported data for all groups except termites. Fresh (“wet”) biomass measurements were converted to dry biomass by applying previously used effective dry weight percentages for key taxonomic groups (*16*) using a default value of 70% water content for groups with missing estimates (especially the soil group "others").

To extend our biomass dataset, we converted measurements of population densities (number of individuals) into biomass densities for soil studies that report only population densities. We based this conversion on the average mass of an individual in each defined taxonomic group (see Fig. 1C) calculated from a wide set of dual measurements, where both abundance and biomass were measured simultaneously. We excluded the group others from this conversion process, because the mass of individuals in this group varies greatly. This conversion was applied only to the soil habitat type, which was divided into the five taxonomic groups of Fig. 1C.

We also standardized the metadata associated with each sample to allow us to integrate the data. This included standardizing the coordinates reported in each study. In cases where the source study did not report the coordinates of the sampling site, we attempted to locate the coordinates of the site from other research that was conducted in the same site or from the most accurate description available. When accurate coordinates or descriptions were not available, we resorted to less-accurate region-based localization (see Supplementary Materials).

The ecological setting of each sample was categorized at the biome level based on the World Wildlife Fund (WWF) Terrestrial Ecoregions Of The World (*44*). To these natural biomes, we added two additional human-associated biomes: pastures and croplands (see fig. S3) (*45*). The surface area of each biome was calculated on the basis of the map that defines the WWF ecoregions, with relative areas converted to pasture and croplands. We combined several biomes of similar natures into “aggregated biomes” to base our estimates on more available data. We combined all tropical and subtropical forests (WWF biomes 1, 2, and 3); all temperate forests (WWF biomes 4 and 5) and temperate grasslands, savannas, and shrublands (WWF biome 8) were combined with montane grasslands and shrublands (WWF biome 10). We excluded flooded grasslands and savannas as well as mangroves (WWF biomes 9 and 14).

### Statistical analysis

To derive estimates of the global biomass and population size of terrestrial arthropods from our harmonized dataset, we analyzed the data in several steps. We stratified samples based on habitat type, biome, and taxonomy (see main text); calculated statistics such as the mean biomass and population densities for each stratified set of samples; and then extrapolated them across the entire extent of that environment.

We aimed to treat each site as a single measurement. A specific locality may contain several different sampling sites if they represent distinct ecosystems within the same biome. For example, a tree-grass mosaic environment includes the tree-associated ecosystem and the grass-associated ecosystem. Each site may contain several samples of the same taxon of arthropods, for example, at different times of the year. To obtain a single estimate per site, we calculated the mean value (biomass or population density) and SD for each taxon in each site and then summed them up for the taxa within each taxonomic group defined above. We arrived at a characteristic value and error for each group within each site. Next, we used bootstrapping (see Supplementary Materials) to average the biomass or population density of each group between the different sites of each biome and arrived at a statistical distribution for the characteristic densities of each group in each biome and habitat type. The means of these distributions in soil are shown by the black circles in Fig. 2. We multiplied the resulting distribution of characteristic biomass or population density by the total area of each biome to arrive at the distribution of the total biomass or population of each group in each biome and habitat type. Last, we summed the various distributions using a Monte Carlo process to arrive at the distribution of total biomass or population in each habitat type, from which we extracted the mean and random errors (the means of these final distributions were almost identical to their medians; the random errors were taken as the 95% confidence interval).

For each taxonomic group in each biome and habitat type, we extracted a 95% confidence interval from the resulting distributions described above (error bars in Fig. 3). The uncertainty ranges reported in the main text and in Fig. 5 were calculated by taking the sum of the lower or upper bounds of the 95% confidence intervals for all such distributions. We used this sum of bounds of the 95% confidence intervals to account for random errors and some possible systematic errors, as this way of summing includes possible correlations between the underlying statistical errors. This procedure increased the uncertainty range by up to a factor of 4 with respect to the random error. As a sensitivity check, we also calculated the above estimates using a range of modified assumptions (grouping and averaging the data in different reasonable ways) and also after removing parts of our data (see Supplementary Materials).

## Supplementary Materials

### This PDF file includes:

Supplementary Text Figs. S1 to S5 Tables S1 to S7 References
