# Supplementary material for: The global biomass and number of terrestrial arthropods
Source: Sci Adv. 2023 Feb 3;9(5):eabq4049. doi: 10.1126/sciadv.abq4049 (PMC9897674; doi:10.1126/sciadv.abq4049)
Supplement: Supplementary file 1 — Supplementary Text Figs. S1 to S5 Tables S1 to S7 References [file sciadv.abq4049_sm.pdf]

Science Advances
AAAS

Supplementary Materials for

The global biomass and number of terrestrial arthropods

Yuval Rosenberg *et al.*

Corresponding author: Ron Milo, ron.milo@weizmann.ac.il

Sci. Adv. **9**, eabq4049 (2023)
DOI: 10.1126/sciadv.abq4049

The PDF file includes:

Supplementary Text
Figs. S1 to S5
Tables S1 to S7
Legend for data S1
References

Other Supplementary Material for this manuscript includes the following:

Data S1

Supplementary Text

Table of Contents

SUPPLEMENTARY TEXT 2

1. SAMPLING YEARS AND LOCATIONS 4

2. DEFINITIONS OF BIOMES 4

3. STATISTICAL ANALYSIS 5

3.1. Data preprocessing 5
3.2. Bootstrapping procedure 5
3.3. Sensitivity analysis 6

4. DETAILED EVALUATION OF THE GLOBAL BIOMASS OF TERMITES 7

4.1. Detailed estimates of termite biomass in global biomes 8
General considerations *8*
Tropical and subtropical forests *8*
Temperate forests *9*
Boreal forests/taiga *9*
Tropical and subtropical grasslands, savannas and shrublands *9*
Temperate grasslands, savannas and shrublands *10*
Mangroves and flooded grasslands and savannas *10*
Tundra *10*
Mediterranean forests, woodlands and scrub *10*
Deserts *11*
Croplands *12*
Pasture *12*
4.2. Comparison with other global termite estimates 12

5. CONSIDERATIONS FOR BIOMASS EVALUATION IN TREE CANOPIES 13

6. SUPPLEMENTARY UPPER ESTIMATE FOR THE BIOMASS OF ABOVE-GROUND ARTHROPODS 13

6.1 Crude upper estimate for the biomass of lepidopterans 14
6.2 Crude upper estimate for the biomass of Orthopterans 15
6.3 Evaluating the biomass of prominent migrating and outbreaking insects 16
6.4 Bounding the mass of arthropods associated with livestock dung 17
6.5 Sanity checks for above-ground arthropods biomass based on trophic interactions 19

7. CONSISTENCY CHECK FOR THE BIOMASS ESTIMATE OF SOIL MACROINVERTEBRATES 19

FIG. S1 20

FIG. S2 21

FIG. S3 22

FIG. S4 23

FIG. S5. 24

TABLE S1. 25

TABLE S2. 27

TABLE S3. 28

TABLE S4. 29

TABLE S5. 30

TABLE S6. 31

TABLE S7. 32

REFERENCES USED 33

1. Sampling years and locations

We track the arthropod sampling times and locations as reported in the literature. Figure S1 depicts the distribution of the sampling dates for soil arthropods for the various taxonomic groups. Figure 1B of the main text depicts the date distribution for all arthropods in both the soil and above ground habitats. Each sampling site receives a weight of one, which is divided over the range of years in which the sampling occurred, according to the amount of reported sampling dates for that site: a single sample date in a single year contributes one count to that year's bar; while 10 repeated samplings composed of 7 measurements in a certain year, and 3 in another year, contributes 0.7 counts to the former year, and 0.3 to the later year. The publication date of the original data was used where we could not determine the sampling date.

The data represent a wide range of years, with significant contributions starting in the late 1960s. The data contain both population measurements and biomass measurements, each having over 300 measurement sites, where in about 200 sites both the population and biomass were measured.

Figure S2 show the ≈300 sampling locations integrated in this study, divided according to the taxonomic groups used. Each location may include several sampling sites, and sample different taxa. Unlike Fig. 1A of the main text, Fig. S2 depicts only soil sampling locations, and presents information on the accuracy of each marked location. In cases where the source study did not report the coordinates of the sampling site, we attempted to locate the coordinates of the site from other research that was conducted in the same site, or from the most accurate description available. We marked these coordinates as 'approximate'. When accurate coordinates or descriptions were not available, we have resorted to less accurate region-based localization. Figure S2 shows the centers of these regions as 'region-level coordinates'.

2. Definitions of biomes

The ecological setting of each sample was categorized at the biome level based on the WWF ecoregion framework, or Terrestrial Ecoregions of the World (*44*). To these natural biomes, we added two additional human-associated biomes - pastures and croplands, as defined by The Food and Agriculture Organization (FAO) (*45*) (see Fig. S3). The surface area of each biome was calculated based on the map that defines the WWF ecoregions, and relative areas converted to pasture and croplands. We combined several biomes of similar nature into 'aggregated biomes' in order to base our estimates on more available data. We combined all tropical and subtropical forests (WWF biomes 1,2,3); all temperate forests (WWF biomes 4,5); temperate grasslands, savannas, and shrublands (WWF biome 8) were combined with montane grasslands and shrublands (WWF biome 10). We also excluded flooded grasslands and savannas, and mangroves (WWF biomes 9,14).

3. Statistical analysis

Here we shortly present the scheme used for extrapolating global estimates from our dataset and statistically testing it. The description focuses on biomass evaluation, but also applies to population evaluation.

3.1. Data preprocessing

The steps taken for data preprocessing were:

1. Convert wet weight to dry weight.
2. Filter out inadequate data.
3. Average the soil data over each reported taxon in each site. This represents averaging samples made at different times, repetitions at similar locations, etc.
4. Sum data for all taxa in each site according to the aggregated groups and data type (biomass density\population density) they are in.
5. Use sites with both biomass and population data to convert population data to biomass density. This is performed using a global average for the weight of an individual taxon (this is not done for the taxa group 'others', as their body masses vary too much). Using an average mass of an individual for each biome results in similar values.

   At this point, we get, for each combination of habitat type, biome, and taxonomic group, a list of biomass density values with their random errors, each corresponding to a single site (one geographic location can have several sites).

   (See Methods in the main text for further details)

3.2. Bootstrapping procedure

1. We continue with the following for each habitat type - biome - taxon combination:
   0. Resample the biomass density values 10^5 times. Each resampling is made with repetitions, where the individual biomass density values are randomly generated from a normal distribution based on the biomass density mean and standard error.
   1. Calculate the arithmetic mean density for each resample.
   2. Use the histograms of the resampled means to extract a density estimate for the probability density functions (PDFs), arithmetic means, and 95% confidence intervals (CIs). Figure S4 shows example histograms for acarians in boreal forests, which have 22 samples; and for termites in temperate forests, which is especially poor in data, having only 6 points that are quite different from one another.

1. To estimate the PDF of the total biomass, we use a Monte-Carlo process: we randomly choose a value from the PDFs of each taxon-biome density means, multiply it by the biome area, and add all the values together for each habitat type (Fig. S5). We repeat this 10^5 times.

2. We extract the median, means, and 95% CI from the PDFs of the total biomass.
3. We also add all the lower / upper values of the CIs of step 1 to create an extended error range. This corresponds to assuming significant correlations between the underlying estimates due to systematic errors etc. This results in a total biomass estimation of 300 Mt, with an uncertainty range of 100-500 Mt.

3.3. Sensitivity analysis

The exact methods used for estimating the global biomass and abundance affect its values. We tested the robustness of our estimates to various changes in the dataset, estimation procedure, and assumptions. Table S2 summarizes this sensitivity analysis and shows that our estimate is quite robust. We emphasize that this does not exclude the existence of possible major biases in our dataset, as described in the discussion of the main text.

We found that removing a single site from our dataset usually has a very small effect on the overall biomass estimate, but in some cases can affect the overall soil biomass estimate by up to about 3%. We also found that removing outliers in our dataset can reduce the overall soil biomass estimate by about 6%. We defined the outliers in log-space, as being at least two standard deviations away from the mean of the data points in each taxonomic group - biome pair that contain more than three data-points.

We also checked the effects of blindly dividing the data of the taxonomic group 'others' according to subphylum, and using only direct biomass measurements (excluding population measurements converted to biomass). We've seen that each such change increases the overall estimate by about 10%, and applying both changes increases the estimate by about 20%. Blindly stratifying data in the taxonomic group 'others' by subphylum creates a bias by averaging each subphylum data over sites with only implicit data for such subphylum. This effectively deletes null values (zeros) for such subphyla where they were not reported for any reason (*46*). Correcting for this bias in the averaging process causes the subphylum-divided data to agree with the stratification used in the main text.

Varying the biogeographic resolution

Our bootstrapping procedures stratify and average the data according to habitat-type, taxonomic group, and biome. We chose this procedure while considering the characteristics of the available data and the bias-variance tradeoff (*31*). However, the biomes used may include regions that are very different from one another, with potentially very different arthropods abundances. This may introduce biases to our estimates. We tested how refining the geographic resolution used in the bootstrapping procedure affects the overall biomass estimate.

We divided the biomes according to well known six biogeographic realms (*47*): the Nearctic, Palearctic, Afrotropical, Indomalayan, Austalasian and Neotropical realms. For this sensitivity analysis, we focused only on a subset of our soil data, where measurements under the same taxonomic group and biome spans over different biogeographic realms. We estimated the total biomass of this subset in the usual way, and compared it to an estimate that divides the data in

each biome into its biogeographic realms during the bootstrapping procedure. This division increased the total biomass estimate of the relevant subset by only about 10%.

Our extended error range includes even some extreme unrealistic assumptions on our dataset. Not stratifying the data according to taxonomy (hence averaging over many partial measurements), resulted in halving the estimate, while treating the entire ice-free land surface as a single biome resulted in doubling the estimate.

The effect of including samples with only macroarthropods on the biomass estimate of the 'others' soil arthropods group

In principle, measurements that included only macroarthropods (30 of the 117 sites with soil biomass data of the group 'others'), might introduce an underestimate. However, the biomass densities measured in these sites are not systematically lower than those that sample the entire arthropods community. Excluding these sites does not significantly change the estimated total biomass in the natural biomes, but will significantly weaken the coverage of the global estimate by removing most of the available data for 'others' in croplands and pasture land, which holds ≈25 Mt biomass.

The effect of including samples where ants and termites were measured together with other soil arthropods

It is possible that some of the data points for ants and for termites originate from measurements that did not account for their unique dispersal, with no special effort to sample their nests. Removing soil data points for ants and termites in sites where additional groups were measured resulted in a reduction of 8 Mt in the global biomass of ants, and an increase of 22 Mt in the global biomass of termites. This is within the uncertainty reported in the main text. Future work should better assess such possible biases, and help reduce the resulting uncertainties.

4. Detailed evaluation of the global biomass of termites

Termites seem to compose about forty percent of the global biomass of arthropods in soil, but measured biomass data is especially scarce. This section estimates the termites' biomass density for each of the global biomes of Fig. S3. It is based on literature from about 140 measurement sites. Geographical trends in the termites' population are used to further divide the biomes where relevant data is available, thus improving our estimates. Three estimates are produced for each biome, based on arithmetic means: the mid estimate is our best estimate for the termites' biomass density, while the high and low estimates serve as upper and lower bounds for the estimation. These bounds reflect different extrapolation assumptions and various errors. It's possible for the true biomass density of various biomes to surpass these bounds due to systematic errors and the scarcity of available data.

Table S5 summarizes our detailed estimates for the various biomes, from which we estimate the total global termite biomass to be about 80 Mt dry weight, with an extended error range between 50 and 120 Mt. This is about 20% lower than the median of our main estimate for termites, but is within its confidence interval (Fig. 4 of the main text).

These estimates should be taken with a grain of salt due to the scarcity of available data, and the

high temporal and spatial variations in biomass density. The systematic errors are made worse by the effects of fragmentation (*48*), termites' high beta-diversity (*33*, *49*), and their endemism that produce intercontinental differences in their biomass (*32*).

4.1. Detailed estimates of termite biomass in global biomes

General considerations

Termites are most abundant in warm, humid areas, such as the tropics (*14*). However, they are strongly affected by floods (*50*). Termites do not exist in cold or cool areas, such as the tundra. They are found roughly between -45 and +45 latitudes.

Their abundance changes after an ecosystem is disturbed, and depends on historical factors rather than the current situation alone (*33*). The current analysis is focused on termites in soils and litter, as the techniques and data on their abundance elsewhere is quite limited (*33*).

We use a conversion ratio of 0.27 from wet to dry biomass weight (*16*). Olson WWF biomes refers to David M. Olson *et.al.* biomes characterization for the World Wildlife Fund (*44*).

Tropical and subtropical forests

This aggregated biome includes the standard WWF biomes 1-3, excluding pasture and crop lands. Tropical rain forests (biome 1) accounts for ≈86% of the total aggregated biome area, while tropical and subtropical dry broadleaf forests (biome 2) accounts for ≈12%, and tropical and subtropical coniferous forests (biome 3) accounts for ≈2% of the total area. Biome 1 also represents ≈90% of the tropical forests sampled data in our dataset. About half of the area is located in the Neotropical realm, while 30% in Indo-Malay, and 15% in Afrotropic. This is also roughly represented by the frequency of the samples in our database.

64 sites were found for this biome, 42 of which had direct biomass estimates. The biomass density spans the range 0-4000 mg/m^2 dry weight. Some measurements from Cameroon (*51*) have exceptionally high values (due to a surge of a very large endemic termite species), which are relaxed to about 4000 mg/m^2 using a log-transformed mean of all similar measurements within the original study (*32*).

The simple arithmetic mean of all available measurements is 1850 mg/m^2 dry weight, or *1280* mg/m^2 dry weight, depending on the value taken for the Cameroon sites.

Sanderson's estimates (*14*) produce an area-weighted sum of 2770 mg/m^2 dry weight for these biomes, disregarding potential underestimates. Following this logic with our updated data and a different way to account for pasture and crop lands results in a similar estimate of about *3100* mg/m^2 dry weight.

Sanderson (*14*) suggested that the true figure for tropical rain forests could be as high as 3000-4000 mg/m^2 dry weight, due to potential undersamplings. This claim is partly supported by some more recent measurements (*32*, *52*, *52*).

We take our mid estimate to be *2200* mg/m^2, as their average.

Temperate forests

Sanderson mentions that the only measurement of termites in temperate forests until 1996 was made in southern Australia's sclerophyll forest (*54*), with 810 mg/m^2 dry weight. We were not able to verify that this measurement was indeed made in a temperate forest, rather than a forest in the biome "Mediterranean forests, woodlands, and scrub or sclerophyll forests" (WWF biome 12). In (*50*), another measurement of about 1350 mg/m^2 dry weight is mentioned from Queensland Australia. We could not verify that this measurement corresponds to a temperate forest. King *et.al.* (*55*) have measured termites biomass in four different eastern US temperate forests, from Connecticut (Lat. 41°57'N) down to northern Florida (Lat. 29°43'N), with much lower average values in the range 0-45.5 mg/m^2 dry weight, from north to south, averaging to about 14 mg/m^2 dry weight. The big difference between the Australian and US measurements means that the way in which we average the two regions greatly changes the final result (being dominated by Australia). The unique features of the Australian ecoregions, and the large beta-diversity and intercontinental biomass differences of termites, suggests we should separate the biome into two main areas - Australia, and the rest of the world.

Using equal weights on all 5 measured high values (0,5,13,91,810) gives an upper estimation of *184* mg/m^2 dry weight. The area of Australasian temperate forests is only a few percent of the global temperate forests area (≈6.7% of the total area is in Australia and New Zealand, and 5.2% from Australia alone), and New Zealand is a home for only three indigenous species, and perhaps few other invasive species (*56*). Using 6% as an approximation for the area with high biomass, and the average of the USA measurements for temperate forests in the rest of the world, we get an average density of *62* mg/m^2 dry weight, which becomes *78* mg/m^2 dry weight if we account also for the measurement from Queensland.

Assuming that the Australian measurements were not representative for this biome and extrapolating the trend found in (*49*) by latitude, we take 8% of the area to contain the northern Florida termites density (rough estimate of the area within similar latitudes as Florida + the Australian areas), while the rest is assigned an average density of 3 mg/m^2 dry weight (the average of 0, 2.5, 6.5 measured in more northern US sites). The lower estimate is thus *6* mg/m^2 dry weight.

Boreal forests/taiga

This biome is too cold for termites, so their biomass there is *0*.

Tropical and subtropical grasslands, savannas and shrublands

The simple average of all 23 data points is 1000 mg/m^2 dry weight, which grows to *1100* mg/m^2 dry weight if we use a single mean value (S01 of (*16*)) for the Ivory coast measurements by (*57*). A Zaire measurement (S15 of (*16*)) was made in a degraded Savannah, and had an exceptionally high biomass due to mound building termites. Removing this single measurement reduces the mean to about *800* mg/m^2 dry weight.

The data seems to be divided into two groups that correspond to arid and non-arid locations: various arid locations have around 200-500 mg/m^2 dry weight, while non-arid locations have about 2500 mg/m^2 dry weight. To estimate the area for each group of measurements, we follow

(*14*). Sanderson's data gives an area-weighted average biomass of 1860 mg/m^2 dry weight. We get *1400* mg/m^2 dry weight using all of our available data, and keeping the same proportions of arid and non-arid areas is kept when removing pasture and crop lands and updating the ecoregion's division.

We hence take our low estimate to be *800* mg/m^2 dry weight, our medium estimate *1100* mg/m^2 dry weight, and our upper estimate *1400* mg/m^2 dry weight.

Temperate grasslands, savannas and shrublands

Montane grasslands and shrublands (WWF biome 10) are too cold for termites, so we take a value of *0* biomass for its area, being 56% of the total aggregated biome area.

We have found only three measurements in temperate grasslands, savannas, and shrublands (biome 8): 1400 ± 460 mg/m^2 dry weight from west Texas (*58*), 220 mg/m^2 dry weight of Heterotermes aureus in a Shrub invaded desert in Arizona (*59*), and 0.6 mg/m^2 dry weight in a Colorado grassland at 40 degrees latitude and next to pasture land (*60*) (also reviewed in (*16*)). This low value could be due to the high latitude, which is quite typical for this biome.

We thus take the upper estimate as *620* mg/m^2 dry weight, assuming all biome 8 has the typical value found in west Texas (following (*14*)). The low estimate of *190* mg/m^2 dry weight assumes such Texas values hold for only about 30% of WWF biome 8, based on latitude, and neglecting other contributions. Our mid estimate of *400* mg/m^2 dry weight is given by the average of the two estimates above.

Mangroves and flooded grasslands and savannas

Flooded areas (WWF biome 9 and 14) are excluded in this study.

Tundra

This biome is too cold for termites, so their biomass there is *0*.

Mediterranean forests, woodlands and scrub

This biome contains estimates from two studies in Australia:
Abensperg-Traun and de Boer (*61*) report on three measurements of subterranean termites in west Australia with 520 ± 50 mg/m^2 dry weight (at a woodland with red soil), 310 ± 40 mg/m^2 dry weight (mallee with yellow soil), and 260 ± 20 mg/m^2 dry weight (heath dominated by shrubs and grasses with sandy yellow soil).

The biomass of mound building termites in this area was measured to be 1000 ± 130 mg/m^2 dry weight for a Wandoo woodland, and 460 ± 70 mg/m^2 dry weight for a Casuarina shrubland (*62*).

While the biogeographic separation used in (*14*) treats only some of these sites as part of the mediterranean biome, the updated database (*44*) includes all of them in biome 12.
The southern Australia's sclerophyll forest (*54*), with 810 mg/m^2 dry weight might also be considered in this biome.

We hence get the mean values of 360 ± 40 mg/m^2 dry weight for subterranean termites and 1100 ± 100 mg/m^2 dry weight for both subterranean and mound building termites. Neglecting the heath and the Casuarina shrubland measurements (*14*) changes the mean values to 420 ± 30 mg/m^2 dry weight and 1400 ± 160 mg/m^2 dry weight, respectively.

This biome is divided to the different biogeographic realms as follows:

AA - Australasia	25%
AT - Afrotropic	3%
PA - Palearctic	63.7%
NA - Nearctic	3.7%
NT - Neotropic	4.6%

We assume mounds are found in between 25% - 35% of the total area, and hence get the high estimate of *845* mg/m^2 dry weight (taking the upper estimates while neglecting the heath and the Casuarina shrubland measurements, and assuming 35% mounds cover), mid estimate of *584* mg/m^2 dry weight (using 30% mound cover), and low estimate of *512* mg/m^2 dry weight (with 25% mound cover and lower estimates).

Deserts

The data for this biome is especially limited, and may be skewed towards locations with relatively high termite abundance. Measurements of subterranean termites in semi-arid African and Australian locations (*14*) typically have a biomass density of ≈270 mg/m^2 dry weight, while mound building termites may account for a significant addition of ≈460 mg/m^2 dry weight. These values should be taken very cautiously, as they may not be representative for the desert biome (see (*14*) and discussion above).

Another study (*63*), from a shrub-invaded desert grassland in Arizona USA, measured the abundance of several foraging termite species, and estimated a total biomass density of 112 mg/m^2 dry weight (This location is part of WWF biome 13, but is regarded by some reviews (*14*, *50*) to be a temperate grassland when considering another study (*59*) by the same authors). This value agrees with the strong correlation between soil moisture and termites biomass in warm regions (*14*, *48*) and refs. therein.

The apparently limited distribution of desert termites to specific habitats within deserts (e.g., (*64*, *65*)) is another indicator for the low average density of desert termites. However, no supporting direct studies were found for the majority of deserts, where actual biomass density could be lower than the above mentioned figures.

Following Sanderson, a value of *700* mg/m^2 dry weight is taken as an upper limit to our density estimate for termites in deserts. A value of *100* mg/m^2 dry weight is taken as a lower limit to our estimate, based on the Arizona shrub-invaded desert (*63*). It is possible that the true figure is lower than this estimate. An intermediate figure of *300* mg/m^2 dry weight is taken as our best estimate based on available data.

Croplands

This biome includes arable land and permanent crops, as defined by the FAO and mapped in (*45*). There are 26 unique sites with samples in croplands, with 25 having a direct biomass measurement.

The arithmetic average for all crop sites is *1160* mg/m^2 dry weight, but there is a significant variability between different crop areas. Our current available data is mostly from tropical regions, so this value is probably an overestimate.

According to (*45*), the crop area is separated between the various biomes as follows: ≈23% within tropical forests, 24% temperate and mixed forests, and most other areas within grasslands, savanna and shrublands (of all kinds).

The average and standard error of termites on crops where tropical forests once stood is 1400 ± 300 mg/m^2 dry weight. On tropical savannah this reduces to 1060 ± 700 mg/m^2 dry weight (based on very limited data). For crops on temperate forests, we take the value range of the natural biome. Also, about 22% are in temperate grasslands and shrublands (the vast majority is grasslands), where we take the biomass values of the natural biome. So if we assume 22% temperate grasslands/savannas, and 31% tropical grasslands/savannas, the average density across all croplands is thus within the range 760 ± 240 mg/m^2 dry weight. We take the mid and low estimates to be *760* mg/m^2 dry weight and *520* mg/m^2 dry weight, respectively.

Pasture

This biome includes permanent pasture, as defined by the FAO and mapped in (*45*). We treat pasture land in a similar way to crop land. The average of all 22 datapoints is *570* mg/m^2 dry weight.

According to (*45*), the pasture area is separated as follows: ≈10% on tropical forests (640 ± 300 mg/m^2), 8% temperate and mixed forests (biome values), ≈20% are in temperate grasslands (3 ± 2 mg/m^2), ≈4% in deserts (biome values), ≈3% Tundra (0 mg/m^2) and the remaining are in tropical and subtropical grassland, savannas and shrublands (470 ± 200 mg/m^2). This gives a total estimate of 340 ± 150 mg/m^2 dry weight. The mid and low estimates are thus *340* mg/m^2 dry weight and *190* mg/m^2 dry weight, respectively.

4.2. Comparison with other global termite estimates

The two most comprehensive global estimates for termite abundance are by Sanderson (*14*) and Sugimoto *et.al.* (*48*).

Table 3 of (*14*) summarises the biomass estimates, with a global total biomass of about *120 Mt* dry mass. This is within the upper end of our estimation range. Using the same data, Tuma *et.al.* (*24*) estimated termites to hold about *100 Mt* dry biomass, based on a slightly different conversion rate between fresh and dry weight.

The total area considered as part of the termites habitat in (*14*) is about 70% of the current study (72.4 10^{12} m^2, compared to ≈10^{14} m^2). The areas of crop and pasture-land differ the most

between the two studies. Also, Sanderson typically uses a single "most appropriate" estimate for each biome, while comparing to other available data. This often assumes that relatively low estimates are due to under-sampling, with the larger values better representing reality.

Sugimoto *et.al.* (*48*) has a much higher estimate, roughly ranging from 140 Mt to 420 Mt, by extrapolating the range of measured local biomass densities. This yields a mean value of about *300 Mt* dry weight, being much larger than our current global estimate. It is stressed that this estimate is based on little data.

5. Considerations for biomass evaluation in tree canopies

A variety of quantitative sampling and collecting methods for canopy arthropods exist (*66*, *67*), but they typically collect and normalize the data in a way that cannot be directly converted to an absolute density with respect to the land area of the forest floor. However, special efforts may provide an area-based density. We found eight such studies (four using insecticide knockdown techniques (*35*, *68–70*), and four using branch clipping techniques (*71–74*)) in the three forested aggregated biomes.
It's challenging to systematically and efficiently sample tree canopies for population density measurements, especially for tall trees. In addition to the scarcity of data, several taxonomic groups and micro-habitats are known to be undersampled in canopies (*75*). These include microarthropods, termites, caterpillars, tree trunks, perched litter, and epiphytes. Even for groups considered to be well sampled it is hard to know the exact sampling efficiency, which depends on environmental conditions and specifics of the sampling methods, such as canopy access. This has large possible implications for the population estimates, as they are possibly dominated by microarthropods. The undersampled taxa and habitats could also significantly affect the estimated biomass, especially in tropical forests, where termites, perched litter, and epiphytes are more common (see *e.g.,(68)*). Additional biases due to the sampling and collection techniques could be even more significant (*35*). The area-based data for arboreal arthropods biomass in tropical forests is based on studies that make special efforts to reduce the undersampling effects, and are considered to show relatively high density estimates (*35*, *68*). These special efforts include the sampling of epiphytes and precision fogging, placing collecting trays along the entire canopy, close to the falling arboreal arthropods.

6. Supplementary upper estimate for the biomass of above-ground arthropods

Our estimate for the above-ground arthropods is uncertain. Biases such as taxonomic under-sampling and low sampling efficiencies result in the possibility of under estimation.
We address this by performing several supplementary analyses to establish an upper range for the total biomass of above-ground arthropods.

First, we account for the low coverage of the natural variability of biomass densities within each biome using the local variability reported within sampled sites. For each biome, we calculate the 97.5^{th} percentile of the biomass density per site. This is done based on the reported standard error, taking two standard errors away from the mean. For cases no standard error is reported we take it to be half the measured value. We then use the maximal value from these calculated densities (Table S6) as an upper range for the average biomass density in the biome. This results in an upper range of ≈150 Mt globally.

Second, we account for possible undersampled taxonomic groups by generating crude upper estimates for their global biomass. We show below that lepidopterans are likely to contribute less than ≈20 Mt globally, and orthopterans less than ≈30 Mt. These key groups are presumed to be undersampled in our data from tropical forests, which rely on canopy fogging, but are generally present in studies from other regions, where most of the data originates from sampling techniques considered to sample these taxa well (see Table S7), such as 'trap and remove' methods (*76*) where arthropods are trapped and removed with their corresponding units of habitat (volume of grassland, tree branches, etc.). The upper estimates above use wide safety margins, extrapolating high density values both globally and beyond their annual presence. This may account for the presence of other taxa that might be under-sampled in our dataset, keeping in mind that different taxa peak at different times and different places, and that some double-counting is in place. Groups such as high-flying insects are estimated to contribute small amounts.

Altogether, our upper range estimate reaches ≈200 Mt dry biomass for above-ground arthropods. This estimate is equivalent to extrapolating the highest observed biomass density for above-ground arthropods, excluding population outbreaks, over the entire tropical, subtropical and temperate regions (density of 2.4 g/m^2 (*35*)). This measurement also produces a high biomass density per area of foliage (about 280 arthropods weighing 275 mg per m^2 leaf area), relative to measurements using other techniques (*35*). We note however that there is a possibility for underestimation in the value of ≈200 Mt, in spite of the wide margins used.

<u>6.1 Crude upper estimate for the biomass of lepidopterans</u>

Our estimate for the global biomass of caterpillars is based on three independent methodologies that sample local population or biomass densities of caterpillars at different sites. The first method used to sample the abundance of caterpillars is systematic tree felling, in which all plants are cut down in a given area, and the number of caterpillars per unit land area is reported (*77–80*). These studies report characteristic population densities of a few caterpillars per meter squared in both temperate and tropical forests.

A second method is based on various forms of branch clipping, where a section of a branch is enclosed with a net to capture all individuals residing on the branch. Studies using this approach (*74*, *80–84*) report population densities or biomass densities per foliage mass or per leaf surface area. Characteristic population and biomass densities from these studies are a few individuals per square meter of leaf area or ≈0.5 milligram of caterpillars per gram of foliage.

Another method is a visual inspection of caterpillars in all plants up to 2 meters along a walking transect (*85*). This data could be converted into an estimate of population density using the area of the walking transect, and expanding the estimate to include all vegetation by assuming uniform vertical distribution of caterpillars across plant canopies, and a total canopy height of 20 meters. Using this approach to convert raw caterpillar counts to population densities yields an estimate of a few individuals per meter squared.

In order to convert these characteristic densities into global estimates of caterpillar biomass, we first convert population densities into biomass densities using characteristic values of caterpillar body weight. We rely on two studies which have sampled two natural communities of caterpillars and measured both the number of individuals and biomass (*86*, *87*). By dividing caterpillar biomass by the total number of individual caterpillars, we arrive at a characteristic value of ≈20 mg of dry body weight per caterpillar. We also used a study which measured the average length of caterpillars (*85*), and converted it into biomass using an allometric model (*88*). This procedure yields a similar estimate. Thus, we use 20 mg dry weight as the characteristic caterpillar body mass. This means that the characteristic population densities of the tree felling and walking transect methods correspond to a biomass density of roughly 50 mg dry weight per meter squared, and that the population density for the branch clipping approach corresponds to about 60 mg per meter square of leaf area.

We upscale the characteristic biomass densities to a global estimate for each of the characteristic values. For biomass densities reported per meter square of land surface, we multiply the densities by 10^{14}, which is roughly the total ice-free surface area. For densities reported per meter square of leaf surface area, we multiply the densities by 2×10^{14}, which is roughly the global total leaf surface area (Bar-On and Milo 2019). For densities reported per gram of foliage, we multiply the density by 3×10^{16}, which is roughly the total dry mass of leaves globally (Bar-On and Milo 2019). This yields an estimate of 5 Mt dry weight for the tree felling and walking transect methods (when extrapolating based on land area), and 10-15 Mt dry weight for the two characteristic densities reported using the branch clipping method. Integrating these different estimates, we estimate a global biomass of ≈10 Mt dry weight for caterpillars.

We take 10 Mt also as an upper estimate for the biomass of adult lepidopterans, which is another group that is underrepresented in our data. This is because most larvae don't survive to adulthood (*34*), with significant mortality continuing through pupation (e.g. due to parasitism), and because lepidopterans lose weight during eclosion (when adults emerge from their pupae) (*89*). Thus, our upper estimate for the global biomass of lepidopterans is ≈20 Mt.

6.2 Crude upper estimate for the biomass of Orthopterans

Orthoptera, especially grasshoppers, were estimated to contribute most of the total arthropod biomass in the grass layer in grassland ecosystems (*90*). Grasshoppers are present in our above-ground dataset, and are assumed to be sampled relatively efficiently by the various trap-remove techniques. However, we have a limited amount of such data, and we can improve our estimate using studies that focus on sampling grasshoppers specifically.

Adult grasshopper densities in western US during peak seasons are estimated at up to 5 individuals per square meter. This is based on the data compiled by USDA-APHIS (*91*) during the years 2011-2021. We assumed mid-range values for each abundance category in the summarizing maps, and as much as 50 individuals per square yard for the top category, "15+". The active season of grasshoppers in western US is shorter than six months, yielding an upper estimate of 2.5 adults per square meter on average. We find that the area of outbreaks (with 15+ ind. per square yard) is about 3% of the total area tested, providing some reference for the

contribution from such outbreaks. Using an adult average body-weight of 100mg (*92*, *93*), this translates to roughly 250 mg/m^2 dry weight. Similar values were found in peak season in grasslands of California (*93*) and Idaho (*94*). In California this translated to a dry biomass density of 40 mg/m^2 on an annual average. We extrapolate the density of 250 mg/m^2 dry weight globally to arrive at an upper estimate of ≈30 Mt dry weight for orthopterans.

6.3 Evaluating the biomass of prominent migrating and outbreaking insects

The population of numerous insect species are known to oscillate dramatically, undergo massive outbreaks, or migrate in large masses (*95*). These populations can defoliate entire areas before their numbers rapidly decline because of unsustainable population densities, or because of change in environmental conditions such as the weather (*95*).
These populations can also easily be missed by random samplings.

High-flying insects that may evade many sampling schemes probably contribute relatively little biomass globally. Few studies have measured the absolute biomass density of flying insects. One study in the UK (*96*) has measured a total annual dry biomass of about one kilo-ton of insects across an area of 70,000 km^2. This corresponds to about 2 Mt of flying insects (annually) when naively extrapolated globally. As a comparison, the world's largest moth or butterfly migrations weigh less than a few hundred dry tons (*96*), and the most massive modern locust swarms can reach tens of kilo-tons (*97*). Certain bark beetles (Coleoptera: Curculionidae: Scolytinae) may also create massive outbreaks, which probably contribute not more than several kilo-tons of biomass (see below).

Data from the Canadian National Forestry (National Forestry Database-Canadian C...) show that insects defoliate roughly 0.04% of Canada forests annually (area of moderate to severe defoliation, including beetle-killed trees). In the US, defoliators alone consume lower percentages of total forested area (excluding bark beetles, *Agrilus planipennis*, and disease) (*98*). Data for grasshoppers in western US (Outbreak and Survey Info : USDA ARS) show severe outbreaks (of more than 15 individuals per square yard) at about 3% of the monitored area. These figures are much lower than the ≈10% foliage defoliation observed regularly (*99–102*). Hence the effect of outbreaks seem to be well within the uncertainty range reported in the main text.

Bounding the biomass of outbreaking bark beetles (Coleoptera: Curculionidae: Scolytinae)

Certain bark beetle populations can increase dramatically during outbreaks, being a major source of forest defoliation in North America. In Western United States roughly 100 million trees were killed per year from *Dendroctonus ponderosae* between the years 1997-2010 (*103*). Assuming a typical ≈1,500 adult beetles and ≈10,000 larvae per dead tree and a lifespan of roughly one year (*104*), we estimate that the US population of bark beetles were roughly one trillion (10^{12}) bark beetles. Using an individual biomass of ≈5 mg dry weight per beetle (*105*), the total biomass in the US during the mentioned outbreak was about 5 kilo-tons. Similar outbreaks occurred also in Western Canada, and to a lesser extent in Europe and Asia (*106*). We thus estimate the global biomass of outbreaking bark beetles to be on the order of kilo-tons, and not more than a few tens of kilo-tons.

6.4 Bounding the mass of arthropods associated with livestock dung

Our data exclude various unique habitats. These include flooded areas, animal waste (from livestock or wild animals), tree bark and trunks, and flying insects (while in air or escaping capture). Animal waste may host a significant amount of arthropod biomass. We show below that this biomass is likely to be less than ≈10 Mt.

Livestock are a dominant contributor to the biomass of large terrestrial animals (*20*), and annually produce about 2 Gt of organic matter in manure. This figure is calculated from the nitrogen inputs of over 100 Mt N from livestock manure (about 100 Mt is annually left on pasture, and roughly 30 Mt is applied to soils) (*36*), together with the C:N ratios between nitrogen contents and organic matter (*107*) (this ratio is about 21 for dairy cattle, 17 for non-dairy cattle, 13 for layer chickens, which we also take for other poultry, 11 for swine, and we approximate the rest as 20). This is equivalent to about 6Mt of organic matter being globally produced every day.

Coprophagous arthropods such as flies and dung beetles play a significant role in decomposing this manure, but their population depends on many environmental conditions and farming practices (*108–112*), such as temperature, moisture, and parasite control. Additional related arthropods, such as attracted predators, represent a small biomass fraction of the dung arthropod communities (e.g.,(*110*)), and hence can be neglected. We use the rate of manure production to evaluate an upper limit for the biomass of manure decomposing arthropods, in three ways:

1. We first use direct biomass measurements of dung feeders in cow pats. Our main data is from summer arthropod communities in pasture cattle dung on a dairy farm near Ithaca, New York (*110*). The measured dry biomass of dung feeders grew to over 12.5% of the initial dry cow pat mass within five days, being about 6% on average at any given moment within these five days. Large dipterans dominated the biomass. If we assume that the growth curves in (*110*) are typical and extrapolate them to the 6Mt of daily produced manure, we find that it holds an average of about 0.4Mt biomass for at least five days. This produces a biomass stock of about (0.4 Mt per day) × (5 days) = 2 Mt inside animal waste. Accounting for the biomass dynamics beyond the reported five days will possibly increase this estimate.

This measured biomass is not necessarily representative. It was taken in a single season and location, and with limited repetitions and variations. Merritt and Anderson (*111*) report a biomass weight that is only one to a few percent of the cow pat weight, using different conditions and techniques. However, their larval development time was longer (mostly up to a month, growth curves were not provided), possibly producing a similar biomass stock evaluation. Both studies followed the arthropod community only in the initial period of dung decomposition, and did not report the reduction in dung mass. This makes it hard to evaluate the potential for the studied cow pats to host more arthropods biomass, beyond the reported times. However, large flies lay their eggs only in the first few hours after dung production (*110*), and typically take days to a month to develop.

Laboratory studies of black soldier fly larvae (Diptera: Stratiomyidae) feeding on manure show similar results - in (*113*), the larvae gained a total biomass of over 10% of the given manure

which lasted for several days. In (*114*), the final larval dry weight was only several percent of the given dry manure, but they took about a month to develop.

2. We estimate the biomass potential, using the lifespan of the decomposing arthropods and their ECI - Efficiency of Converting Ingested food to body substance (*115*), which is similar to Lindeman's trophic efficiency (*116*). The data in (*110*) suggests that the ECI calculated from dry biomass and dry food (manure) is larger than 12.5% for dung-feeders. Others (*115*, *117*) gave typical ECI values of 10-30% for various insects, mostly herbivores; and studies on black soldier fly larvae found ECI of about 12% from cow manure (*118*), 12-16% from swine manure (*119*), 4-8% from poultry manure (*120*), and about 3% ECI from these manures after drying them and reducing their nutritional values (*121*). Similar low values were reported for Aphodius larva in dung (*117*). Since we are interested in estimating an upper bound for the biomass of manure decomposing arthropods, we assume the ECI to be 20%, resulting in up to about 400 Mt of total annual biomass dry weight. The above studies show a typical larval lifespan of a few days to about a month (being highly dependent on many factors including temperature and moisture, see e.g.,(*122*) and the references therein), resulting in an upper limit of about 30 Mt dry biomass stock (the result of dividing the annual 400 Mt biomass by 12 months).

3. We can make a sanity check by comparing the estimates above with the relative consumption rate, or consumption index (*115*) - the amount of daily ingested food per larva, relative to its average body weight. If we assume that the animal waste is not accumulating over time, and that arthropods ingest all of it, we find that larvae with a total biomass of 2-30 Mt dry weight consume the daily manure flux containing 6 Mt organic matter if on average each larva consumes 0.2 - 3 times its body weight per day. This agrees with data from other insects (*117*), and from laboratory studies on black soldier fly larvae feeding on manure (*113*, *114*) (the latter have a relative consumption rate larger than one).

The unrealistic assumption that arthropods ingest all of the produced manure is made since these biomass estimates only serve as an order-of-magnitude upper bound. Arthropods are not the only manure decomposers. Some of the manure is decomposed by worms, bacteria, fungi, and even large animals; and some undergo weathering and physical breaking. However, some arthropods feed on the decomposing bacteria and fungi, and these decomposers can be effectively considered as part of the process of arthropods dung decomposition.

While many adult dung-feeders were not directly considered here, their inclusion will probably not affect the estimates by much. Adult dipterans, for instance, were not measured by the referenced studies, since they live outside the manure. However, arthropods typically have a high mortality rate during their larval stage, where they also gain their maximal weight. Hence, the larval biomass roughly represents the biomass of the entire population.

We conclude an upper estimate of ≈10 Mt for the standing dry biomass of dung-related arthropods, where the actual value might be much lower than that.

6.5 Sanity checks for above-ground arthropods biomass based on trophic interactions

Sanity checks based on trophic interactions agree with the above estimates. Globally, leaves constitute ≈30 Gt of dry biomass, which is mainly found in forests (*123*). Assuming that leaf biomass turns over roughly every two years (*124*) and that arthropods consume about 10% of them (*99–102*), we get that herbivorous arthropods consume roughly 1.5 dry Gt per year. Since the efficiency of converting ingested food into biomass in herbivorous immature arthropods is on average less than 20% (*117*), we find that herbivory generates about 300 Mt of dry arthropods per year. The lifespan of most insects is shorter than a few months (*125*), which yields an average stock of up to ≈100 Mt dry biomass of arthropods feeding on leaves on earth.

Another sanity check is based on the rate of consumption of terrestrial arthropods by key consumers. Previous studies (*126*) have estimated that insectivorous birds consume roughly 150 Mt dry biomass of arthropods annually, while ants might consume a similar amount. Spiders were estimated to consume roughly 120-250 Mt dry biomass annually. Limited data for animals such as lizards, frogs and shrews, suggest that they might consume large arthropod quantities in some areas (*126*). However, they are considered to exert low predation pressures in other areas, such that their total global consumption is probably lower than that of birds, spiders or ants (*126*). Hence, the total *annual* arthropods prey biomass is of the order of 400-500 Mt dry weight. This total value includes both above-ground and soil arthropods, and is similar to the annual herbivores secondary productivity estimated above. Both of these sanity checks agree with our estimated stocks of above-ground arthropods.

7. Consistency check for the biomass estimate of soil macroinvertebrates

We compare our estimates with a recent comprehensive study of the abundance of soil macroarthropods (*17*) and find general agreement with our biomass estimate for the group ‘others’. To compare our results and (*17*), we have considered only arthropod counts in (*17*), excluding earthworms. The general methodology used by (*17*) to sample the abundance of termites and ants may underestimate their true abundances, by missing colonies (*21*, *50*), or sampling only parts of the communities such as only foraging ants (*18*). Thus, we also exclude these groups from the analysis. The remaining taxa are broadly consistent with the ‘others’ group in our definitions of soil arthropods, and we use our estimate of the total biomass of this group as a reference for comparison. To compare the population densities in (*17*) to our estimates we convert population densities to biomass densities and extrapolate them globally. We rely on several studies that utilize the same sampling methodology as in (*17*), but measured both population and wet biomass densities for each taxon (*127–130*). We estimate the characteristic wet body weight of each taxon by summing the reported biomass densities of each taxon from all studies and dividing them by the corresponding sum of reported population densities. Finally, we estimate the global biomass of soil macroarthropods (excluding ants and termites) by multiplying the characteristic population densities for each taxon in (*17*) by their calculated characteristic body weights, converting it to dry weight assuming 70% water content by weight, multiplying the resulting biomass densities by the ice-free land surface area ($\approx 1.3\times10^{14}$ m^2), and summing the resulting global biomass for all taxa. This analysis yields an estimate of ≈75 Mt, which is similar to our estimate of ≈50 Mt for the group ‘others’. This comparison is not a rigorous analysis of the data reported in (*17*), and is based on some strong simplifying assumptions. Nevertheless, it supports the consistency of our estimates with the published literature.

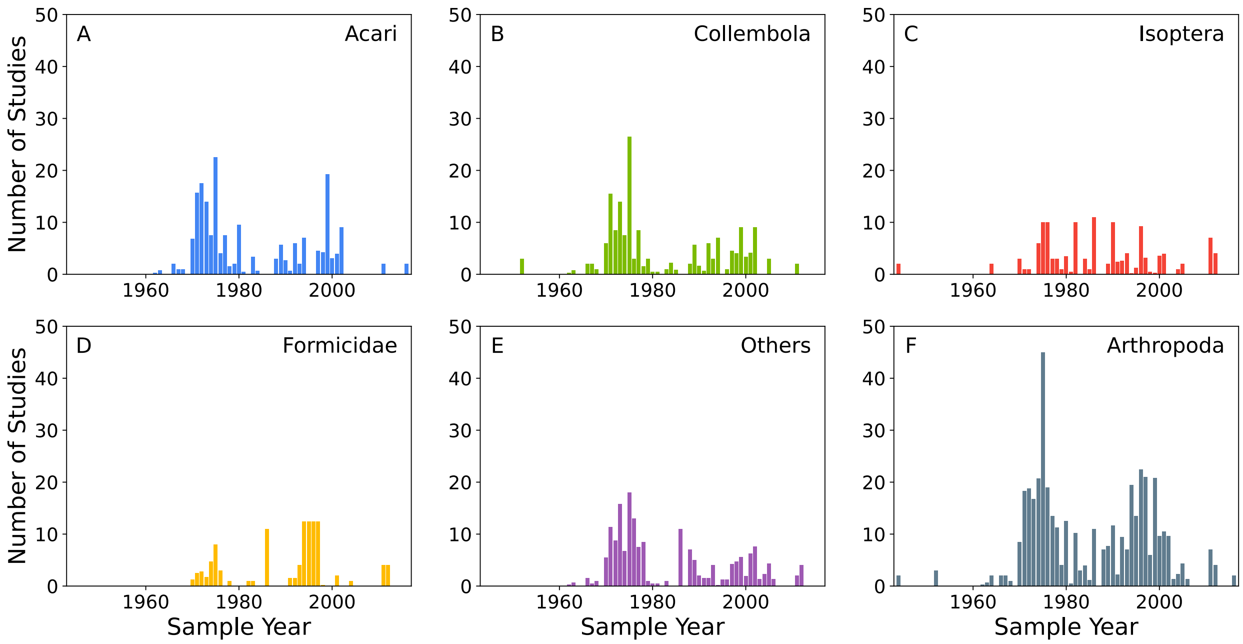

Fig. S1.

Sampling year distribution of the soil arthropods abundance measurements used in this study. (A-E) Divided to the various taxonomic groups we used. (F) For all soil arthropods. Figure 1**B** of the main text also includes data for the 'Above-ground' habitat type.

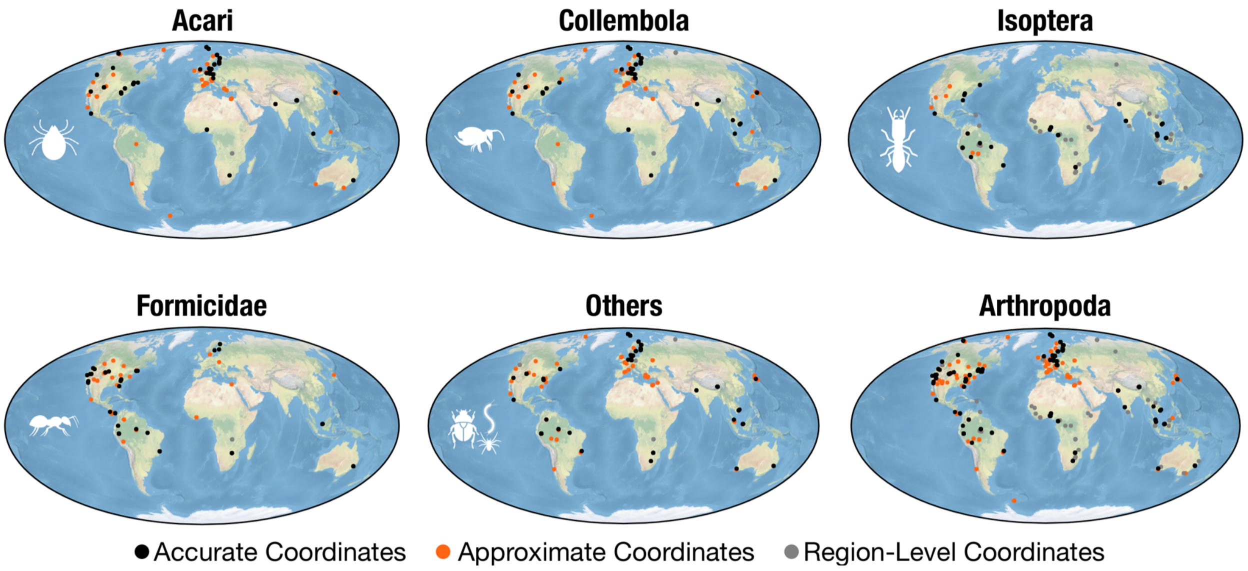

Fig. S2.

Sampling locations of soil arthropods abundance measurements used in this study. Divided by taxonomic groups (subplots) and level of location accuracy (marker color).

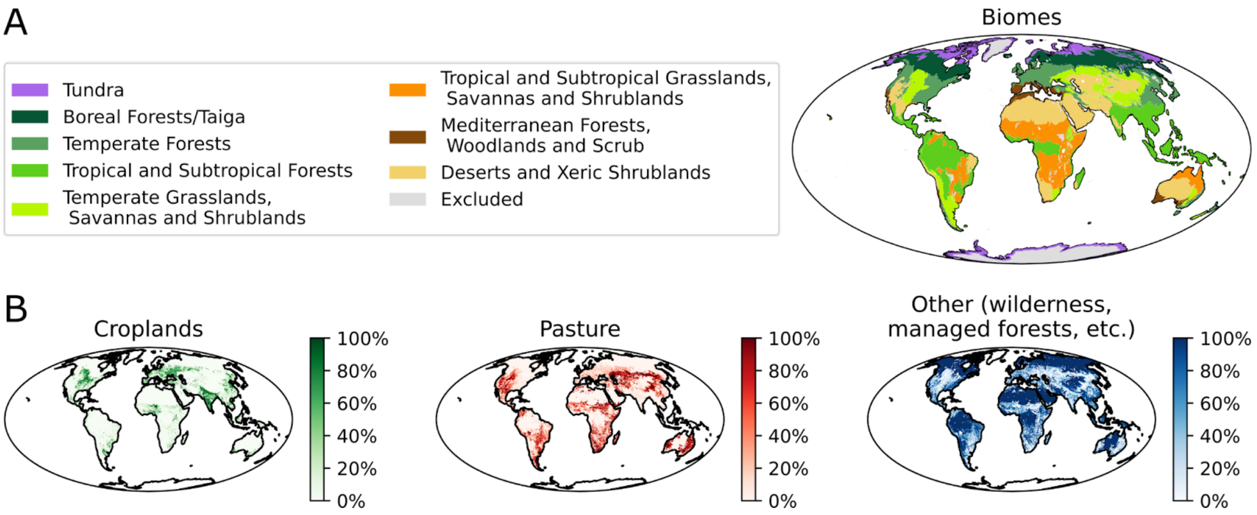

Fig. S3.

The geographic definition of the different biomes used in this study. (A) The geographic range of the different aggregated biomes used in this study, except croplands and pastures. (B) The fraction of land surface at each location covered by croplands, pasture or other land (such as wilderness, managed forests etc.). We removed the surface area of croplands and pastures from each biome, and defined them as two separate biomes.

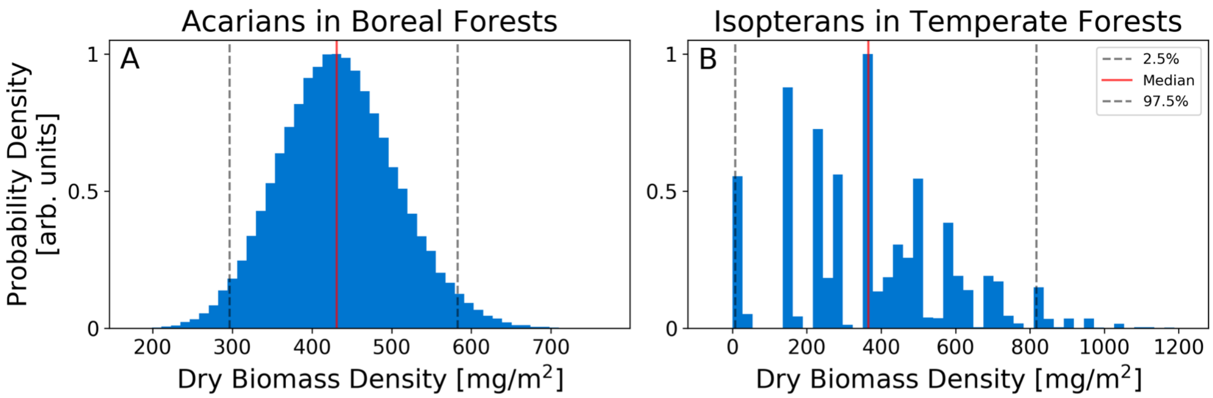

Fig. S4.

Illustrative examples of estimated biomass probability density functions (PDFs) calculated from a bootstrapping process. (A) For mites in boreal forests, and (B) for termites in temperate forests, which have especially limited data.

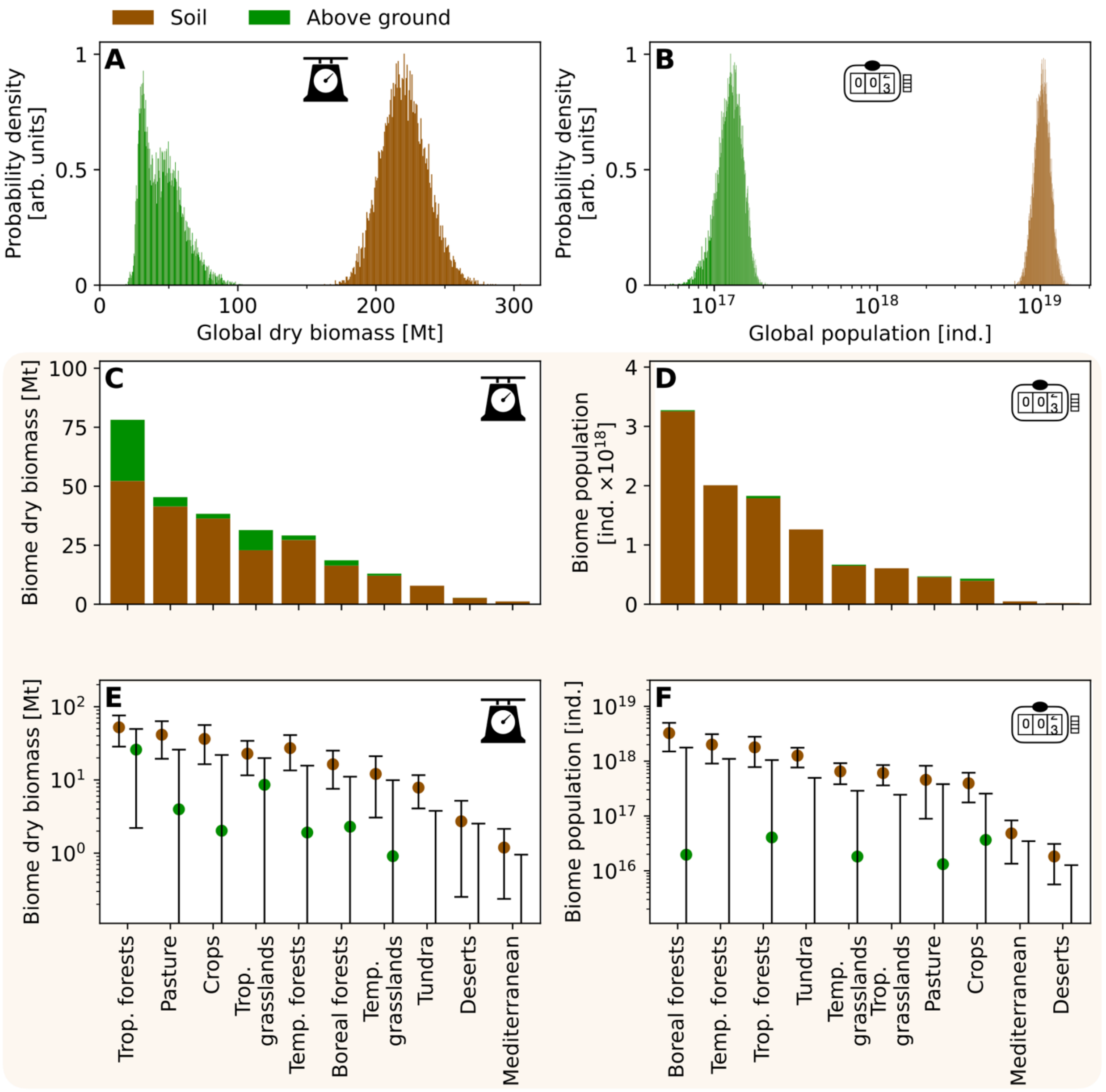

Fig. S5.

Total biomass and number of individuals - globally and across biomes. Estimated probability density functions in the two habitat types: (A) For the total global biomass, and (B) for the total global population (in logarithmic scale). Calculated from a Monte-Carlo process of our bootstrapped data (see text). For each biome: (C) and (E) show the total dry biomass on logarithmic and linear scales, respectively. (D) and (F) show the same for total populations. Black errors bars represent 95% confidence intervals. Biomes are as defined above, but with shortened names. We take the global uncertainty range as the sum of the lower or upper bounds of all biome-level uncertainties.

Table S1.
A detailed description of our dataset.

Column name	Description
ID	A unique identification number for the measurement-based estimate.
site	A unique name for the site, typically as described in the literature.
coordinates	Coordinates as given in the literature.
country	Country where the sampling took place.
biome	Sampled biome, as reported in the literature.
standard biome	Biome as defined by the WWF Terrestrial Ecoregions Of The World (37).
aggregated biome	Sampled biome, according to the definitions in the section Definitions of biomes. Based on coordinates and description in reference.
environment	Sampled habitat type (e.g., soil, above-ground, canopy).
aggregated environment	A unified habitat type: soil and litter, above ground, or canopy. (Above ground and canopy are later merged)
taxon	Sampled taxon or taxa group, as reported in the literature.
family, super-family, order, sub-class, class, sub-phylum, phylum	Various taxonomic ranks of the sampled taxa.

(table continues on next page)

Table S1.
A detailed description of our dataset (continued).

Column name	Description
numerical value	Numerical value measured, in the units specified in 'units' column. If only a range is given in the literature, the numerical value is its mean.
standard deviation	Standard deviation as reported in the literature. If only a range is given in the literature, the data in the standard deviation column equals half the range.
units	Units of the numerical value and standard deviation.
reference	Reference to the study from which the data was extracted. Typically expressed as the first author and year of publication.
link (doi)	Link to the reference.
source in text	The location in the referenced text, where the extracted data can be found.
date	Date of the sampling or publication.
remarks	Additional remarks, such as the accuracy of the coordinates.
studied group	The taxonomic groups studied in the reference.
synthetic coordinates	Approximate coordinates of the sampling location if precise location is unknown. Based on the text as described in 'remarks'.
sampling technique	The sampling techniques used for samples made above-ground.

Table S2.

Relative change in total soil estimate under various changes in the analyses. Rounded to a single significant digit.

	Relative change
Removing a single measurements site	-1% to +3%
Removing taxon-biome 'outliers'	-6%
Using only direct biomass measurements	+10%
Blindly stratifying 'others' by subphylum	+10%
Stratifying data in each biome by biogeographic realms	+10%
Detailed biomass evaluation for termites	-10%

Table S3.

Summary of biomass density data presented in Fig. 2A of the main text. Mean biomass density (mg/m^2), 95% confidence interval (in brackets), and number of sites with relevant data (N), for each biome and taxonomic group. Data is rounded to a single significant digit.

Biome	Acari	Collembola	Formicidae	Isoptera	Other
Boreal Forests / Taiga	400 (300-600)<br>N = 22	500 (100-1000)<br>N = 21	5 (1-8)<br>N = 2	0<br>N = 1	200 (100-300)<br>N = 22
Crops	100 (40-200)<br>N = 7	30 (10-40)<br>N = 4	70 (30-100)<br>N = 8	1000 (800-2000)<br>N = 26	700 (200-2000)<br>N = 14
Deserts and Xeric Shrublands	4 (2-7)<br>N = 7	1 (0.2-3)<br>N = 6	60 (10-100)<br>N = 5	80 (0.4-200)<br>N = 3	0 (0-0)<br>N = 4
Mediterranean Forests, Woodlands and Shrublands	80 (20-200)<br>N = 12	40 (20-70)<br>N = 8	40<br>N = 1	600 (60-1000)<br>N = 4	0 (0-0)<br>N = 12
Pasture	40 (9-90)<br>N = 14	20 (2-40)<br>N = 12	100 (60-200)<br>N = 14	800 (400-1000)<br>N = 29	500 (300-800)<br>N = 20
Temperate Forests	800 (500-1000)<br>N = 53	300 (200-500)<br>N = 50	300 (50-800)<br>N = 25	400 (7-800)<br>N = 6	800 (500-1000)<br>N = 47
Temperate Grasslands, Savannas and Shrublands	300 (200-400)<br>N = 18	200 (100-300)<br>N = 19	300 (50-800)<br>N = 7	700 (0.6-2000)<br>N = 2	700 (200-1000)<br>N = 12
Tropical and Subtropical Forests	200 (100-300)<br>N = 9	50 (30-70)<br>N = 13	300 (70-700)<br>N = 25	2000 (1000-3000)<br>N = 63	600 (300-1000)<br>N = 25
Tropical and Subtropical Grasslands, Savannas and Shrublands	70 (30-100)<br>N = 2	10<br>N = 2	800 (400-1000)<br>N = 7	1000 (600-2000)<br>N = 23	100 (7-300)<br>N = 9
Tundra	200 (100-300)<br>N = 51	400 (200-700)<br>N = 40	0<br>N = 2	0<br>N = 2	50 (10-100)<br>N = 18

Table S4.

Summary of population density data presented in Fig. 2B of the main text. Mean abundance density (individuals/m^2), 95% confidence interval (in brackets), and number of sites with relevant data (N), for each biome and taxonomic group. Data is rounded to single significant digits.

Biome	Acari	Collembola	Formicidae	Isoptera	Other
Boreal Forests / Taiga	100000 (80000-200000) N = 17	80000 (20000-200000) N = 17	8 (2-10) N = 2	0 N = 1	1000 (700-2000) N = 22
Crops	20000 (8000-30000) N = 7	4000 (3000-7000) N = 4	300 (100-400) N = 8	2000 (500-4000) N = 10	1000 (300-2000) N = 12
Deserts and Xeric Shrublands	700 (200-1000) N = 6	200 (20-500) N = 5	4 N = 1	3 (0.6-4) N = 2	40 (20-60) N = 4
Mediterranean Forests, Woodlands and Shrublands	20000 (4000-30000) N = 12	8000 (4000-10000) N = 8		100 (30-300) N = 2	7000 (200-20000) N = 12
Pasture	7000 (2000-10000) N = 7	7000 (300-20000) N = 5	300 (100-600) N = 14	1000 (600-3000) N = 27	200 (50-500) N = 18
Temperate Forests	100000 (60000-200000) N = 18	70000 (20000-200000) N = 18	300 (10-900) N = 5	6 (0.8-20) N = 4	20000 (6000-40000) N = 28
Temperate Grasslands, Savannas and Shrublands	70000 (40000-100000) N = 4	50000 (30000-70000) N = 8	1000 (100-4000) N = 4	2 N = 1	1000 (70-3000) N = 10
Tropical and Subtropical Forests	90000 (40000-200000) N = 8	10000 (8000-20000) N = 11	700 (300-1000) N = 14	2000 (1000-2000) N = 53	700 (400-1000) N = 22
Tropical and Subtropical Grasslands, Savannas and Shrublands	50000 (30000-60000) N = 2	7000 (2000-10000) N = 2	1000 (600-1000) N = 7	1000 (700-2000) N = 19	800 (200-2000) N = 8
Tundra	40000 (30000-60000) N = 51	70000 (30000-100000) N = 38	0 N = 1	0 N = 1	500 (200-900) N = 17

Table S5.

Summary of detailed termite biomass analysis for the various biomes. Biomass density data shows mid estimates, with low and high estimates in brackets.

Biome	Biomass Density [mg/m^2 dry weight]	Total area [10^{12} m^2]	Total biomass [dry Mt]
Boreal Forests/Taiga	0	14.9	0
Crops	750 (500 - 1200)	15.2	11
Deserts and Xeric Shrublands	300 (100 - 700)	19.6	6
Mediterranean Forests, Woodlands and Scrub	600 (500 - 850)	1.60	1
Pasture	350 (200 - 600)	27.7	10
Temperate Forests	80 (5 - 200)	10.6	1
Temperate Grasslands, Savannas and Shrublands	400 (200 - 600)	5.61	2
Tropical and Subtropical Forests	2200 (1300 - 3100)	17.0	37
Tropical and Subtropical Grasslands, Savannas and Shrublands	1100 (800 - 1400)	10.9	12
Tundra	0	11.7	0
World Total		135	80

Table S6.
The upper estimate of biomass density across biomes.

Aggregated biome	Upper dry biomass density [mg/m^2]
Boreal Forests/Taiga	540
Crops	530
Deserts and Xeric Shrublands	23
Pasture	810
Temperate Forests	1110
Temperate Grasslands, Savannas and Shrublands	870
Tropical and Subtropical Forests	4700
Tropical and Subtropical Grasslands, Savannas and Shrublands	1890

Table S7.

Number of sites in our database with biomass measurements of above-ground arthropods per sampling technique and biome. 'Trap and remove' corresponds to various methods intended to remove all arthropods within a unit of habitat they inhabit, such as quickly removing a volume of grassland or branch-clipping.

Aggregated biome	Canopy fogging	Hand capture + Sweep nets	'Trap and remove'	Vacuum suction
Boreal Forests/Taiga			6	
Crops				13
Deserts and Xeric Shrublands		5	1	
Pasture			7	
Temperate Forests			7	
Temperate Grasslands, Savannas and Shrublands			8	
Tropical and Subtropical Forests	2			
Tropical and Subtropical Grasslands, Savannas and Shrublands			3	

References used

*Our dataset is based on references (*14*, *16*, *19*, *32*, *33*, *35*, *50–52*, *55*, *60*, *65*, *68–74*, *93*, *102*, *130–196*).

Data S1. (separate file)
The raw dataset of measured local abundance densities and metadata.

REFERENCES AND NOTES

1. S. L. Pimm, C. N. Jenkins, R. Abell, T. M. Brooks, J. L. Gittleman, L. N. Joppa, P. H. Raven, C. M. Roberts, J. O. Sexton, The biodiversity of species and their rates of extinction, distribution, and protection. *Science* **344**, 1246752 (2014).

2. G. Ceballos, P. R. Ehrlich, A. D. Barnosky, A. García, R. M. Pringle, T. M. Palmer, Accelerated modern human–induced species losses: Entering the sixth mass extinction. *Sci. Adv.* **1**, e1400253 (2015).

3. S. N. Stuart, J. S. Chanson, N. A. Cox, B. E. Young, A. S. L. Rodrigues, D. L. Fischman, R. W. Waller, Status and trends of amphibian declines and extinctions worldwide. *Science* **306**, 1783–1786 (2004).

4. K. V. Rosenberg, A. M. Dokter, P. J. Blancher, J. R. Sauer, A. C. Smith, P. A. Smith, J. C. Stanton, A. Panjabi, L. Helft, M. Parr, P. P. Marra, Decline of the North American avifauna. *Science* **366**, 120–124 (2019).

5. G. Ceballos, P. R. Ehrlich, R. Dirzo, Biological annihilation via the ongoing sixth mass extinction signaled by vertebrate population losses and declines. *Proc. Natl. Acad. Sci. U.S.A.* **114**, E6089–E6096 (2017).

6. P. Eggleton, The state of the world's insects. *Annu. Rev. Env. Resour.* **45**, 61–82 (2020).

7. M. L. Forister, E. M. Pelton, S. H. Black, Declines in insect abundance and diversity: We know enough to act now. *Conservat. Sci. Prac.* **1**, e80 (2019).

8. D. L. Wagner, Insect declines in the anthropocene. *Annu. Rev. Entomol.* **65**, 457–480 (2020).

9. R. van Klink, D. E. Bowler, K. B. Gongalsky, A. B. Swengel, A. Gentile, J. M. Chase, Meta-analysis reveals declines in terrestrial but increases in freshwater insect abundances. *Science* **368**, 417–420 (2020).

10. M. S. Crossley, A. R. Meier, E. M. Baldwin, L. L. Berry, L. C. Crenshaw, G. L. Hartman, D. Lagos-Kutz, D. H. Nichols, K. Patel, S. Varriano, W. E. Snyder, M. D. Moran, No net insect

abundance and diversity declines across US long term ecological research sites. *Nat. Ecol. Evol.* **4**, 1368–1376 (2020).

11. C. J. Macgregor, J. H. Williams, J. R. Bell, C. D. Thomas, Moth biomass has fluctuated over 50 years in Britain but lacks a clear trend. *Nat. Ecol. Evol.* **3**, 1645–1649 (2019).

12. F. Sánchez-Bayo, K. A. G. Wyckhuys, Further evidence for a global decline of the entomofauna. *Austral. Entomol.* **60**, 9–26 (2021).

13. J. A. Noriega, J. Hortal, F. M. Azcárate, M. P. Berg, N. Bonada, M. J. I. Briones, I. Del Toro, D. Goulson, S. Ibanez, D. A. Landis, M. Moretti, S. G. Potts, E. M. Slade, J. C. Stout, M. D. Ulyshen, F. L. Wackers, B. A. Woodcock, A. M. C. Santos, Research trends in ecosystem services provided by insects. *Basic Appl. Ecol.* **26**, 8–23 (2018).

14. M. G. Sanderson, Biomass of termites and their emissions of methane and carbon dioxide: A global database. *Global Biogeochem. Cycles* **10**, 543–557 (1996).

15. J. E. Losey, M. Vaughan, The economic value of ecological services provided by insects. *Bioscience* **56**, 311 (2006).

16. H. Petersen, M. Luxton, A comparative analysis of soil fauna populations and their role in decomposition processes. *Oikos* **39**, 288–388 (1982).

17. P. Lavelle, J. Mathieu, A. Spain, G. Brown, C. Fragoso, E. Lapied, A. De Aquino, I. Barois, E. Barrios, M. E. Barros, J. C. Bedano, E. Blanchart, M. Caulfield, Y. Chagueza, J. Dai, T. Decaëns, A. Dominguez, Y. Dominguez, A. Feijoo, P. Folgarait, S. J. Fonte, N. Gorosito, E. Huerta, J. J. Jimenez, C. Kelly, G. Loranger, R. Marchão, R. Marichal, C. Praxedes, L. Rodriguez, G. Rousseau, L. Rousseau, N. Ruiz, C. Sanabria, J. C. Suarez, J. E. Tondoh, A. De Valença, S. J. Vanek, J. Vasquez, E. Velasquez, E. Webster, C. Zhang, Soil macroinvertebrate communities: A world-wide assessment. *Glob. Ecol. Biogeogr.* **31**, 1261–1276 (2022).

18. P. Schultheiss, S. S. Nooten, R. Wang, M. K. L. Wong, F. Brassard, B. Guénard, The

abundance, biomass, and distribution of ants on Earth. *Proc. Natl. Acad. Sci. U.S.A.* **119**, e2201550119 (2022).

19. N. Fierer, M. S. Strickland, D. Liptzin, M. A. Bradford, C. C. Cleveland, Global patterns in belowground communities. *Ecol. Lett.* **12**, 1238–1249 (2009).

20. Y. M. Bar-On, R. Phillips, R. Milo, The biomass distribution on Earth. *Proc. Natl. Acad. Sci. U.S.A.* **115**, 6506–6511 (2018).

21. C. J. Krebs, Ecological methodology. *Addison* (1999).

22. S. Roy, M. M. Roy, R. Bano, P. Saxena, "Soil Microarthropods: Biodiversity and role in grassland and agroforestry ecosystems" in *Agroforestry*, J. C. Dagar, V. P. Tewari, Eds. (Springer, 2017), pp. 669–689.

23. C. B. Williams, The range and pattern of insect abundance. *Am. Nat.* **94**, 137–151 (1960).

24. J. Tuma, P. Eggleton, T. M. Fayle, Ant-termite interactions: An important but under-explored ecological linkage. *Biol. Rev. Camb. Philos. Soc.* **95**, 555–572 (2020).

25. A. M. Potapov, C. A. Guerra, J. van den Hoogen, A. Babenko, B. C. Bellini, M. P. Berg, S. L. Chown, L. Deharveng, Ľ. Kováč, N. A. Kuznetsova, J.-F. Ponge, M. B. Potapov, D. J. Russell, D. Alexandre, J. M. Alatalo, J. I. Arbea, I. Bandyopadhyay, V. Bernava, S. Bokhorst, T. Bolger, G. Castaño-Meneses, M. Chauvat, T.-W. Chen, M. Chomel, J. Cortet, P. Čuchta, A. M. de la Pedrosa, S. S. D. Ferreira, C. Fiera, J. Filser, O. Franken, S. Fujii, E. G. Koudji, M. Gao, B. Gendreau-Berthiaume, D. F. Gomez-Pamies, M. Greve, I. Tanya Handa, C. Heiniger, M. Holmstrup, P. Homet, M. Ivask, C. Janion-Scheepers, M. Jochum, S. Joimel, B. C. S. Jorge, E. Jucevica, L. C. I. de Oliveira Filho, O. Klauberg-Filho, D. Baretta, E. J. Krab, A. Kuu, E. C. A. de Lima, D. Lin, A. Liu, J.-Z. Lu, M. J. Luciañez, M. T. Marx, M. M. McCary, M. A. Minor, T. Nakamori, I. Negri, R. Ochoa-Hueso, J. G. Palacios-Vargas, M. M. Pollierer, P. Querner, N. Raschmanová, M. I. Rashid, L. J. Raymond-Léonard, L. Rousseau, R. A. Saifutdinov, S. Salmon, E. J. Sayer, N. Scheunemann, C. Scholz, J. Seeber, Y. B. Shveenkova, S. K. Stebaeva, M. Sterzynska, X. Sun, W. I. Susanti, A. A. Taskaeva, M. P.

Thakur, M. A. Tsiafouli, M. S. Turnbull, M. N. Twala, A. V. Uvarov, L. A. Venier, L. A. Widenfalk, B. R. Winck, D. Winkler, D. Wu, Z. Xie, R. Yin, D. Zeppelini, T. W. Crowther, N. Eisenhauer, S. Scheu, Globally invariant metabolism but density-diversity mismatch in springtails. *bioRxiv* 01.07.475345 (2022). https://doi.org/10.1101/2022.01.07.475345.

26. H. M. André, X. Ducarme, P. Lebrun, Soil biodiversity: Myth, reality or conning? *Oikos* **96**, 3–24 (2002).

27. S. J. Phillips, M. Dudík, J. Elith, C. H. Graham, A. Lehmann, J. Leathwick, S. Ferrier, Sample selection bias and presence-only distribution models: Implications for background and pseudo-absence data. *Ecol. Appl.* **19**, 181–197 (2009).

28. A. P. Møller, M. D. Jennions, Testing and adjusting for publication bias. *Trends Ecol. Evol.* **16**, 580–586 (2001).

29. C. J. Lortie, L. W. Aarssen, A. E. Budden, J. K. Koricheva, R. Leimu, T. Tregenza, Publication bias and merit in ecology. *Oikos* **116**, 1247–1253 (2007).

30. J. L. Gressitt, Insect biogeography. *Annu. Rev. Entomol.* **19**, 293–321 (1974).

31. T. Hastie, R. Tibshirani, J. Friedman, *The Elements of Statistical Learning: Data Mining, Inference, and Prediction* (Springer New York, ed. 2, 2017).

32. C. A. L. Dahlsjö, C. L. Parr, Y. Malhi, H. Rahman, P. Meir, D. T. Jones, P. Eggleton, First comparison of quantitative estimates of termite biomass and abundance reveals strong intercontinental differences. *J. Trop. Ecol.* **30**, 143–152 (2014).

33. P. Eggleton, “Global patterns of termite diversity” in *Termites: Evolution, Sociality, Symbioses, Ecology*, T. Abe, D. E. Bignell, M. Higashi, Eds. (Springer Netherlands, 2000), pp. 25–51.

34. D. L. Wagner, A. C. Hoyt, “On Being a caterpillar: Structure, function, ecology, and behavior” in *Caterpillars in the Middle: Tritrophic Interactions in a Changing World*, R. J. Marquis, S. Koptur, Eds. (Springer International Publishing, 2022), pp. 11–62.

35. R. J. Dial, M. D. F. Ellwood, E. C. Turner, W. A. Foster, Arthropod abundance, canopy structure, and microclimate in a Bornean lowland tropical rain Forest1. *Biotropica.* **38**, 643–652 (2006).

36. Food and Agriculture Organization of the United Nations (FAO), FAOSTAT 2021; http://www.fao.org/faostat/en/#data/QA.

37. S. F. Sakagami, H. Fukuda, Life tables for worker honeybees. *Res. Popul. Ecol.*. **10**, 127–139 (1968).

38. E. P. on P. P. P. A. T. R. (ppr), EFSA Panel on Plant Protection Products and their Residues (PPR), Scientific Opinion on the science behind the development of a risk assessment of Plant Protection Products on bees (*Apis mellifera*, Bombusspp. and solitary bees). *EFSA Journal*. (2012), vol. 10 p. 2668.

39. N. Hrassnigg, K. Crailsheim, Differences in drone and worker physiology in honeybees (*Apis mellifera*). *Apidologie* **36**, 255–277 (2005).

40. C. G. Jones, J. H. Lawton, M. Shachak, "Organisms as ecosystem engineers" in *Ecosystem Management*, F. B Samson, F. L. Knop, Eds. (Springer New York, 1994), pp. 130–147.

41. S. Seibold, W. Rammer, T. Hothorn, R. Seidl, M. D. Ulyshen, J. Lorz, M. W. Cadotte, D. B. Lindenmayer, Y. P. Adhikari, R. Aragón, S. Bae, P. Baldrian, H. B. Varandi, J. Barlow, C. Bässler, J. Beauchêne, E. Berenguer, R. S. Bergamin, T. Birkemoe, G. Boros, R. Brandl, H. Brustel, P. J. Burton, Y. T. Cakpo-Tossou, J. Castro, E. Cateau, T. P. Cobb, N. Farwig, R. D. Fernández, J. Firn, K. S. Gan, G. González, M. M. Gossner, J. C. Habel, C. Hébert, C. Heibl, O. Heikkala, A. Hemp, C. Hemp, J. Hjältén, S. Hotes, J. Kouki, T. Lachat, J. Liu, Y. Liu, Y.-H. Luo, D. M. Macandog, P. E. Martina, S. A. Mukul, B. Nachin, K. Nisbet, J. O'Halloran, A. Oxbrough, J. N. Pandey, T. Pavlíček, S. M. Pawson, J. S. Rakotondranary, J.-B. Ramanamanjato, L. Rossi, J. Schmidl, M. Schulze, S. Seaton, M. J. Stone, N. E. Stork, B. Suran, A. Sverdrup-Thygeson, S. Thorn, G. Thyagarajan, T. J. Wardlaw, W. W. Weisser, S. Yoon, N. Zhang, J. Müller, The contribution of insects to global forest deadwood decomposition. *Nature* **597**, 77–81 (2021).

42. J. van den Hoogen, S. Geisen, D. Routh, H. Ferris, W. Traunspurger, D. A. Wardle, R. G. M. de Goede, B. J. Adams, W. Ahmad, W. S. Andriuzzi, R. D. Bardgett, M. Bonkowski, R. Campos-Herrera, J. E. Cares, T. Caruso, L. de Brito Caixeta, X. Chen, S. R. Costa, R. Creamer, J. Mauro da Cunha Castro, M. Dam, D. Djigal, M. Escuer, B. S. Griffiths, C. Gutiérrez, K. Hohberg, D. Kalinkina, P. Kardol, A. Kergunteuil, G. Korthals, V. Krashevska, A. A. Kudrin, Q. Li, W. Liang, M. Magilton, M. Marais, J. A. R. Martín, E. Matveeva, E. H. Mayad, C. Mulder, P. Mullin, R. Neilson, T. A. D. Nguyen, U. N. Nielsen, H. Okada, J. E. P. Rius, K. Pan, V. Peneva, L. Pellissier, J. C. P. da Silva, C. Pitteloud, T. O. Powers, K. Powers, C. W. Quist, S. Rasmann, S. S. Moreno, S. Scheu, H. Setälä, A. Sushchuk, A. V. Tiunov, J. Trap, W. van der Putten, M. Vestergård, C. Villenave, L. Waeyenberge, D. H. Wall, R. Wilschut, D. G. Wright, J.-I. Yang, T. W. Crowther, Soil nematode abundance and functional group composition at a global scale. *Nature* **572**, 194–198 (2019).

43. A. Sayers, Serial – Tips and tricks in performing a systematic review. *Br. J. Gen. Prac.* **57**, 999 (2007).

44. D. M. Olson, E. Dinerstein, E. D. Wikramanayake, N. D. Burgess, G. V. N. Powell, E. C. Underwood, J. A. D'amico, I. Itoua, H. E. Strand, J. C. Morrison, C. J. Loucks, T. F. Allnutt, T. H. Ricketts, Y. Kura, J. F. Lamoreux, W. W. Wettengel, P. Hedao, K. R. Kassem, Terrestrial ecoregions of the world: A new map of life on earth. *Bioscience* **51**, 933 (2001).

45. N. Ramankutty, A. T. Evan, C. Monfreda, J. A. Foley, Farming the planet: 1. Geographic distribution of global agricultural lands in the year 2000. *Global Biogeochem. Cycles* **22**, GB1003 (2008).

46. A. Blasco-Moreno, M. Pérez-Casany, P. Puig, M. Morante, E. Castells, What does a zero mean? Understanding false, random and structural zeros in ecology. *Methods Ecol. Evol.* **10**, 949–959 (2019).

47. M. D. F. Udvardy, *A classification of the biogeographical provinces of the world* (IUCN, 1975).

48. A. Sugimoto, D. E. Bignell, J. A. MacDonald, “Global impact of termites on the carbon cycle and atmospheric trace gases” in *Termites: Evolution, Sociality, Symbioses, Ecology*, T. Abe, D. E. Bignell, M. Higashi, Eds. (Springer Netherlands, 2000), pp. 409–435.

49. P. Eggleton, P. H. Williams, K. J. Gaston, Explaining global termite diversity: Productivity or history? *Biodivers. Conserv.* **3**, 318–330 (1994).

50. D. E. Bignell, P. Eggleton, “Termites in ecosystems” in *Termites: Evolution, Sociality, Symbioses, Ecology*, T. Abe, D. E. Bignell, M. Higashi, Eds. (Springer Netherlands, 2000), pp. 363–387.

51. P. Eggleton, D. E. Bignell, W. A. Sands, N. A. Mawdsley, J. H. Lawton, T. G. Wood, N. C. Bignell, The diversity, abundance and biomass of termites under differing levels of disturbance in the Mbalmayo forest reserve, Southern Cameroon. *Philos. Trans. R. Soc. Lond. B Biol. Sci.* **351**, 51–68 (1996).

52. A. E. Anichkin, N. V. Belyaeva, I. G. Dovgobrod, Y. B. Shveenkova, A. V. Tiunov, Soil microarthropods and macrofauna in monsoon tropical forests of Cat Tien and Bi Dup-Nui Ba National Parks, southern Vietnam. *Biol. Bull.* **34**, 498–506 (2007).

53. C. Martius, Occurrence, body mass and biomass of Syntermes spp. (Isoptera: Termitidae) in Reserva Ducke, Central Amazonia. *Acta Amazon.* **28**, 319–319 (1998).

54. K. E. Lee, T. G. Wood, *Termites and soils* (Academic Press, 1971).

55. J. R. King, R. J. Warren, M. A. Bradford, Social insects dominate eastern US temperate hardwood forest macroinvertebrate communities in warmer regions. *PLOS ONE* **8**, e75843 (2013).

56. J. Bain, M. J. Jenkin, Kalotermes banksiae, Glyptotermes brevicornis, and other termites (Isoptera) in New Zealand. *Null* **7**, 365–371 (1983).

57. G. Josens, "Les termites de la savane de Lamto" in *Analyse d’un Ecosystème Tropical Humide: la Sarane de Lamto (Côte d'Ivoire)* (1974), vol. 5, pp. 91–131.

58. M. C. Bodine, D. N. Ueckert, Effect of desert termites on herbage and litter in a shortgrass ecosystem in west Texas. *Rangeland Ecol. Manage.* **28**, 353–358 (1975).

59. M. I. Haverty, W. L. Nutting, J. P. Lafage, Density of colonies and spatial distribution of foraging territories of the desert subterranean termite, heterotermes aureus (Snyder) 123. *Environ. Entomol.* **4**, 105–109 (1975).

60. J. E. Lloyd, R. Kumar, R. R. Grow, J. W. Leethman, V. Keith, Abundance and biomass of soil macroinvertebrates of the Pawnee site collected from pastures subjected to different grazing presures, irrigation and/or nitrogen fertilization, 1970–1971. *Technical report (US International Biological Program Grassland Biome); no 239* (1973).

61. M. Abensperg-Traun, E. S. Boer, Species abundance and habitat differences in biomass of subterranean termites (Isoptera) in the wheatbelt of Western Australia. *Austral. Ecol.* **15**, 219–226 (1990).

62. H. C. Park, J. D. Majer, R. J. Hobbs, Influence of vegetation and soil types on the wheatbelt termite, *Drepanotermes tamminensis* (Hill), in the Western Australian wheatbelt. *Ecol. Res.* **9**, 151–158 (1994).

63. M. I. Haverty, W. L. Nutting, Density, dispersion, and composition of desert termite foraging populations and their relationship to superficial dead wood 1. *Environ. Entomol.* **4**, 480–486 (1975).

64. A. H. Kaschef, L. S. El-Sherif, Distribution of four termite species in the A. *R. Egypt. Insectes Soc.* **18**, 227–232 (1971).

65. A. Shama, Dominance value and community production of desert Arthropoda in Qatar. *Qatar Univ. Sci. J.* **18**, 137–153 (1999).

66. Y. Basset, V. Novotny, S. E. Miller, R. L. Kitching, Methodological advances and limitations in canopy entomology. *Arthropods of tropical forests: Spatio-temporal dynamics and resource use in the canopy*, (2003) pp. 7–16.

67. Y. Basset, N. D. Springate, H. P. Aberlenc, G. Delvare, A review of methods for sampling arthropods in tree canopies, in *Canopy Arthropods,* N. E. Stork, J. Adis, R. K. Didham, Eds. (London, Chapman and Hall, 1996) p. 35

68. M. D. F. Ellwood, W. A. Foster, Doubling the estimate of invertebrate biomass in a rainforest canopy. *Nature* **429**, 549–551 (2004).

69. C. M. P. Ozanne, M. R. Speight, C. Hambler, H. F. Evans, Isolated trees and forest patches: Patterns in canopy arthropod abundance and diversity in *Pinus sylvestris* (Scots Pine). *For. Ecol. Manage* **137**, 53–63 (2000).

70. J. Simandl, Canopy arthropods on Scots pine: Influence of season and stand age on community structure and the position of sawflies (Diprionidae) in the community. *For. Ecol. Manage* **62**, 85–98 (1993).

71. T. D. Schowalter, D. A. Crossley, "Canopy arthropods and their response to forest disturbance" in *Forest Hydrology and Ecology at Coweeta,* W. T. Swank, D. A. Crossley, Eds. (Springer New York, 1988), pp. 207–218.

72. R. A. Werner, Biomass, density, and nutrient content of plant arthropods in the taiga of Alaska. *Can. J. For. Res.* **13**, 729–739 (1983).

73. T. D. Schowalter, Canopy arthropod community structure and herbivory in old-growth and regenerating forests in western Oregon. *Can. J. For. Res.* **19**, 318–322 (1989).

74. N. Hijii, Y. Umeda, M. Mizutani, Estimating density and biomass of canopy arthropods in coniferous plantations: An approach based on a tree-dimensional parameter. *For. Ecol. Manage* **144**, 147–157 (2001).

75. Y. Basset, *Plant Ecol.* **153**, 87–107 (2001).

76. A. L. Turnbull, C. F. Nicholls, A "quick trap" for area sampling of arthropods in grassland communities. *J. Econ. Entomol.* **59**, 1100–1104 (1966).

77. T. J. S. Whitfeld, V. Novotny, S. E. Miller, J. Hrcek, P. Klimes, G. D. Weiblen, Predicting tropical insect herbivore abundance from host plant traits and phylogeny. *Ecology* **93**, S211–S222 (2012).

78. C. L. Seifert, G. P. A. Lamarre, M. Volf, L. R. Jorge, S. E. Miller, D. L. Wagner, K. J. Anderson-Teixeira, V. Novotný, Vertical stratification of a temperate forest caterpillar community in eastern North America. *Oecologia* **192**, 501–514 (2020).

79. O. Mottl, P. Fibich, P. Klimes, M. Volf, R. Tropek, K. Anderson-Teixeira, J. Auga, T. Blair, P. Butterill, G. Carscallen, E. Gonzalez-Akre, A. Goodman, O. Kaman, G. P. A. Lamarre, M. Libra, M. E. Losada, M. Manumbor, S. E. Miller, K. Molem, G. Nichols, N. S. Plowman, C. Redmond, C. L. Seifert, J. Vrana, G. D. Weiblen, V. Novotny, Spatial covariance of herbivorous and predatory guilds of forest canopy arthropods along a latitudinal gradient. *Ecol. Lett.* **23**, 1499–1510 (2020).

80. M. Volf, P. Klimeš, G. P. A. Lamarre, C. M. Redmond, C. L. Seifert, T. Abe, J. Auga, K. Anderson-Teixeira, Y. Basset, S. Beckett, P. T. Butterill, P. Drozd, E. Gonzalez-Akre, O. Kaman, N. Kamata, B. Laird-Hopkins, M. Libra, M. Manumbor, S. E. Miller, K. Molem, O. Mottl, M. Murakami, T. Nakaji, N. S. Plowman, P. Pyszko, M. Šigut, J. Šipoš, R. Tropek, G. D. Weiblen, V. Novotny, Quantitative assessment of plant-arthropod interactions in forest canopies: A plot-based approach. *PLOS ONE* **14**, e0222119 (2019).

81. I. Abbott, T. Burbidge, M. Williams, P. Heurck, Arthropod fauna of Jarrah (*Eucalyptus marginata*) foliage in Mediterranean forest of Western Australia: Spatial and temporal variation in abundance, biomass, guild structure and species composition. *Austral Ecol.* **17**, 263–274 (1992).

82. E. B. Morrison, C. A. Lindell, Birds and bats reduce insect biomass and leaf damage in tropical forest restoration sites. *Ecol. Appl.* **22**, 1526–1534 (2012).

83. P. Banko, R. Peck, S. Yelenik, E. Paxton, F. Bonaccorso, K. Montoya-Aiona, D. Foote, "Dynamics and ecological consequences of the 2013–2014 Koa moth outbreak at Hakalau Forest National Wildlife Refuge" (Tech. Rep. HCSU-058, Hawaii Cooperative Studies Unit,

2014).

84. D. A. Crossley, C. S. Gist, W. W. Hargrove, L. S. Risley, T. D. Schowalter, T. R. Seastedt, "Foliage consumption and nutrient dynamics in canopy insects" in *Forest Hydrology and Ecology at Coweeta*, W. T. Swank, D. A. Crossley, Eds. (Springer New York, 1988), pp. 193–205.

85. D. H. Janzen, "Heterogeneity of potential food abundance for tropical small land birds" in *Migrant Birds in the Neotropics: Ecology, Behavior, Distribution, and Conservation*, A. Keast, E. S. Morton, Eds. (Smithsonian Institution Press, Washington DC, 1980).

86. A. Schuldt, M. Baruffol, H. Bruelheide, S. Chen, X. Chi, M. Wall, T. Assmann, Woody plant phylogenetic diversity mediates bottom-up control of arthropod biomass in species-rich forests. *Oecologia* **176**, 171–182 (2014).

87. H. S. Zandt, A comparison of three sampling techniques to estimate the population size of caterpillars in trees. *Oecologia* **97**, 399–406 (1994).

88. C. W. Wardhaugh, Estimation of biomass from body length and width for tropical rainforest canopy invertebrates. *Aust. J. Entomol.* **52**, 291–298 (2013).

89. F. Molleman, J. Javoiš, T. Esperk, T. Teder, R. B. Davis, T. Tammaru, Sexual differences in weight loss upon eclosion are related to life history strategy in Lepidoptera. *J. Insect Physiol.* **57**, 712–722 (2011).

90. T. Gardiner, J. Hill, D. Chesmore, Review of the methods frequently used to estimate the abundance of orthoptera in grassland ecosystems. *J. Insect Conserv.* **9**, 151–173 (2005).

91. Outbreak and survey info (USDA ARS, 2022); www.ars.usda.gov/plains-area/sidney-mt/northern-plains-agricultural-research-laboratory/pest-management-research/pmru-docs/grasshoppers-their-biology-identification-and-management/outbreak-and-survey-info/outbreak-and-survey-info/

92. L. G. Putnam, E. G. Peters, The growth characteristics, in terms of live weight, of some

grasshoppers (Orthoptera: Acrididae) of Western Canada. *Can. Entomol.* **92**, 908–910 (1960).

93. E. E. Porter, R. A. Redak, H. Elizabeth Braker, Density, biomass, and diversity of grasshoppers (Orthoptera: Acrididae) in a California native grassland. *Great Basin Nat.* **56**, 10 (1996).

94. D. J. Fielding, M. A. Brusven, Historical analysis of grasshopper (Orthoptera: Acrididae) population responses to climate in southern Idaho, 1950–1980. *Environ. Entomol.* **19**, 1786–1791 (1990).

95. G. K. Himes Boor, C. B. Schultz, E. E. Crone, W. F. Morris, Mechanism matters: The cause of fluctuations in boom–Bust populations governs optimal habitat restoration strategy. *Ecol. Appl.* **28**, 356–372 (2018).

96. G. Hu, K. S. Lim, N. Horvitz, S. J. Clark, D. R. Reynolds, N. Sapir, J. W. Chapman, Mass seasonal bioflows of high-flying insect migrants. *Science* **354**, 1584–1587 (2016).

97. A. Steedman, *Locust handbook* (Natural Resources Institute, 1990).

98. Forest Health Protection Publications: Annual Major Forest Insect and Disease Conditions in the United States (USDA Forest Service, 2022); www.fs.usda.gov/foresthealth/publications/fhp/index.shtml.

99. T. D. Schowalter, W. W. Hargrove, D. A. Crossley Jr., Herbivory in forested ecosystems. *Annu. Rev. Entomol.*, **31**, 177–196 (1986).

100. P. D. Coley, J. A. Barone, Herbivory and plant defenses in tropical forests. *Annu. Rev. Ecol. Syst.* **27**, 305–335 (1996).

101. K. S. S. Nair, *Tropical Forest Insect Pests: Ecology, Impact, and Management* (Cambridge Univ. Press, 2009).

102. R. I. Van Hook Jr., Energy and nutrient dynamics of spider and orthopteran populations in

a grassland ecosystem. *Ecol. Monogr.* **41**, 1–26 (1971).

103. A. J. H. Meddens, J. A. Hicke, C. A. Ferguson, Spatiotemporal patterns of observed bark beetle-caused tree mortality in British Columbia and the western United States. *Ecol. Appl.* **22** 1876–1891 (2012).

104. R. T. Graham, L. A. Asherin, M. A. Battaglia, T. Jain, S. A. Mata, Mountain pine beetles: A century of knowledge, control attempts, and impacts central to the Black Hills. *Gen. Tech. Rep.* **353**, 193 (2016).

105. M. Graf, M. L. Reid, B. H. Aukema, B. S. Lindgren, Association of tree diameter with body size and lipid content of mountain pine beetles. *Can. Entomol.* **144**, 467–477 (2012).

106. T. Hlásny, P. Krokene, A. Liebhold, C. Montagné-Huck, J. Müller, H. Qin, K. Raffa, M.-J. Schelhaas, R. Seidl, M. Svoboda, H. Viiri, Living with bark beetles: Impacts, outlook and management options (From Science to Policy, 2019), p. 52.

107. J. Ronald Miner, R. J. Smith, *Livestock Waste Management with Pollution Control* (Midwest Plan Service, 1975).

108. L. Strong, Avermectins: A review of their impact on insects of cattle dung. *Bull. Entomol. Res.* **82**, 265–274 (1992).

109. S. A. Beynon, M. Peck, D. J. Mann, O. T. Lewis, Consequences of alternative and conventional endoparasite control in cattle for dung-associated invertebrates and ecosystem functioning. *Agric. Ecosyst. Environ.* **162**, 36–44 (2012).

110. I. Valiela, Composition, food webs and population limitation in dung arthropod communities during invasion and succession. *Am. Midl. Nat.* **92**, 370–385 (1974).

111. R. W. Merritt, J. R. Anderson, The effects of different pasture and rangeland ecosystems on the annual dynamics of insects in cattle droppings. *Hilgardia* **45**, 31–70 (1977).

112. Food and Agriculture Organization of the United Nations (FAO), Guidelines resistance

management and integrated parasite control in ruminants (FAO, 2004).

113. C. D. Miranda, J. A. Cammack, J. K. Tomberlin, Mass production of the black soldier Fly, *Hermetia illucens* (L.), (Diptera: Stratiomyidae) reared on three manure types. *Animals*, **10**, 1243 (2020).

114. H. M. Myers, J. K. Tomberlin, B. D. Lambert, D. Kattes, Development of black soldier fly (Diptera: Stratiomyidae) larvae fed dairy manure. *Environ. Entomol.* **37**, 11–15 (2014).

115. G. P. Waldbauer, "The consumption and utilization of food by insects" in *Advances in Insect Physiology*, J. W. L. Beament, J. E. Treherne, V. B. Wigglesworth, Eds. (Academic Press, 1968), vol. 5, pp. 229–288.

116. R. L. Lindeman, The trophic-dynamic aspect of ecology. *Ecology* **23**, 399–417 (1942).

117. F. Slansky, J. M. Scriber, Selected bibliography and summary of quantitative food utilization by immature insects. *Bull. Entomol. Soc. Am.* **28**, 43–56 (1982).

118. Q. Li, L. Zheng, N. Qiu, H. Cai, J. K. Tomberlin, Z. Yu, Bioconversion of dairy manure by black soldier fly (Diptera: Stratiomyidae) for biodiesel and sugar production. *Waste Manag.* **31**, 1316–1320 (2011).

119. L. Newton, C. Sheppard, D. W. Watson, G. Burtle, R. Dove, Using the black soldier fly, *Hermetia illucens*, as a value-added tool for the management of swine manure. *Animal and Poultry Waste Management Center, North Carolina State University, Raleigh, NC.* **17**, 18 (2005).

120. D. Craig Sheppard, G. Larry Newton, S. A. Thompson, S. Savage, A value added manure management system using the black soldier fly. *Bioresour. Technol.* **50**, 275–279 (1994).

121. D. G. A. B. Oonincx, D. G. A. Oonincx, A. van Huis, J. J. A. van Loon, Nutrient utilisation by black soldier flies fed with chicken, pig, or cow manure. *J. Insects Food Feed* **1**, 131–139 (2015).

122. J. Couret, E. Dotson, M. Q. Benedict, Temperature, larval diet, and density effects on development rate and survival of *Aedes aegypti* (Diptera: Culicidae). *PLOS ONE* **9**, e87468 (2014).

123. Y. M. Bar-On, R. Milo, The global mass and average rate of rubisco. *Proc. Natl. Acad. Sci. U.S.A.* **116**, 4738–4743 (2019).

124. N. Dong, I. J. Wright, J. M. Chen, X. Luo, H. Wang, T. F. Keenan, N. G. Smith, I. C. Prentice, Rising CO_2 and warming reduce global canopy demand for nitrogen. *New Phytol.* **235**, 1692–1700 (2022).

125. J. R. Carey, Insect biodemography. *Annu. Rev. Entomol.* **46**, 79–110 (2001).

126. M. Nyffeler, Ç. H. Şekercioğlu, C. J. Whelan, Insectivorous birds consume an estimated 400–500 million tons of prey annually. *Sci. Nat.* **105**, 47 (2018).

127. I. M. C. Mboukou-Kimbatsa, F. Bernhard-Reversat, J. J. Loumeto, Change in soil macrofauna and vegetation when fast-growing trees are planted on savanna soils. *For. Ecol. Manage.* **110**, 1–12 (1998).

128. T. Decaëns, P. Lavelle, J. J. J. Jimenez, G. Escobar, G. Rippstein, Impact of land management on soil macrofauna in the Oriental Llanos of Colombia. *Eur. J. Soil Biol.* **30**, 157–168 (1994).

129. G. G. Brown, A. G. Moreno, I. Barois, C. Fragoso, P. Rojas, B. Hernández, J. C. Patrón, Soil macrofauna in SE Mexican pastures and the effect of conversion from native to introduced pastures. *Agric. Ecosyst. Environ.* **103**, 313–327 (2004).

130. E. Blanchart, C. Villenave, A. Viallatoux, B. Barthès, C. Girardin, A. Azontonde, C. Feller, Long-term effect of a legume cover crop (*Mucuna pruriens* var. *utilis*) on the communities of soil macrofauna and nematofauna, under maize cultivation, in southern Benin. *Eur. J. Soil Biol.* **42**, S136–S144 (2006).

131. V. Huhta, A. Koskenniemi, Numbers, biomass and community respiration of soil

invertebrates in spruce forests at two latitudes in Finland. *Ann. Zool. Fennici.* **12**, 164–182 (1975).

132. B. Axelsson, U. Lohm, T. Persson, Enchytraeids, lumbricids and soil arthropods in a northern deciduous woodland: A quantitative study. *Holarct. Ecol.* **7**, 91–103 (1984).

133. C. S. Gist, D. A. Crossley, The litter arthropod community in a southern appalachian hardwood forest: Numbers, biomass and mineral element content. *Am. Midl. Nat.* **93**, 107–122 (1975).

134. J. B. Byzova, A. V. Uvarov, A. D. Petrova, Seasonal changes in communities of soil invertebrates in tundra ecosystems of Hornsund, Spitsbergen. *Pol. Polar Res.*, 245–266 (1995).

135. V. Huhta, M. Räty, Soil animal communities of planted birch stands in central Finland. *Silva Fennica* **39**, 5–19 (2005).

136. T. Persson, E. Bååth, M. Clarholm, H. Lundkvist, B. E. Söderström, B. Sohlenius, Trophic structure, biomass dynamics and carbon metabolism of soil organisms in a scots pine forest. *Ecol. Bull.* **32**, 419–459 (1980).

137. M. Kaspari, M. D. Weiser, Energy, taxonomic aggregation, and the geography of ant abundance. *Ecography* **35**, 65–72 (2012).

138. K. Niijima, Effects of outbreak of the train millipede *Parafontaria laminata armigera* Verhoeff (Diplopoda: Xystodesmidae) on litter decomposition in a natural beech forest in Central Japan. 1. Density and biomass of soil invertebrates. *Ecol. Res.* **13**, 41–53 (1998).

139. V. Huhta, Soil macroarthropod communities in planted birch stands in comparison with natural forests in central Finland. *Appl. Soil Ecol.* **20**, 199–209 (2002).

140. S. Scheu, D. Albers, J. Alphei, R. Buryn, U. Klages, S. Migge, C. Platner, J.-A. Salamon, The soil fauna community in pure and mixed stands of beech and spruce of different age: Trophic structure and structuring forces. *Oikos* **101**, 225–238 (2003).

141. J. P. Curry, Above-ground arthropod fauna of four swedish cropping systems and its role in carbon and nitrogen cycling. *J. Appl. Ecol.* **23**, 853–870 (1986).

142. J. Tsukamoto, J. Sabang, Soil macro-fauna in an Acacia mangium plantation in comparison to that in a primary mixed dipterocarp forest in the lowlands of Sarawak Malaysia. *Pedobiologia* **49**, 69–80 (2005).

143. P. Lavelle, B. Pashanasi, Soil macrofauna and land management in Peruvian Amazonia (Yurimaguas, Loreto). *Pedobiologia* **33**, 283–291 (1989).

144. A. J. Y. Blumberg, P. F. Hendrix, D. A. Crossley, Effects of nitrogen source on arthropod biomass in no-tillage and conventional tillage grain sorghum agroecosystems. *Environ. Entomol.* **26**, 31–37 (1997).

145. T. Rosswall, T. Persson, U. Lohm, Energetical significance of the annelids and arthropods in a Swedish Grassland soil. *Ecol. Bull.*, 1–211 (1977).

146. M. E. Mispagel, E. L. Sleeper, Density and biomass of surface-dwelling macroarthropods in the Northern mojave desert. *Environ. Entomol.* **12**, 1851–1857 (1983).

147. J. F. McBrayer, J. M. Ferris, L. J. Metz, C. S. Gist, B. W. Cornaby, Y. Kitazawa, T. Kitazawa, J. G. Wernz, G. W. Krantz, H. Jensen, Decomposer invertebrate populations in U.S. forest biomes. *Pedobiologia.* **17**, 89–96 (1977).

148. J. G. Palacios-Vargas, G. Castaño-Meneses, J. A. Gómez-Anaya, A. Martínez-Yrizar, B. E. Mejía-Recamier, J. Martínez-Sánchez, Litter and soil arthropods diversity and density in a tropical dry forest ecosystem in Western Mexico. *Biodivers. Conserv.* **16**, 3703–3717 (2007).

149. J. Perner, C. Wytrykush, A. Kahmen, N. Buchmann, I. Egerer, S. Creutzburg, N. Odat, V. Audorff, W. W. Weisser, Effects of plant diversity, plant productivity and habitat parameters on arthropod abundance in montane European grasslands. *Ecography*. **28**, 429–442 (2005).

150. G. K. Douce, Biomass of soil mites (Acari) in Arctic Coastal Tundra. *Oikos* **27**, 324–330 (1976).

151. S.-A. Bengtson, A. Fjellberg, T. Solhöy, Abundance of tundra arthropods in Spitsbergen. *Insect Syst. Evol.* **5**, 137–142 (1974).

152. F. Rodà, J. Retana, C. A. Gracia, C. L. Lange, M. M. Caldwell, G. Heldmaier, J. Bellot, O. L. Lange, H. A. Mooney, U. Sommer, *Ecology of Mediterranean Evergreen Oak Forests* (Springer Science and Business Media, 1999).

153. H. G. T. Ndagurwa, J. S. Dube, D. Mlambo, M. Mawanza, The influence of mistletoes on the litter-layer arthropod abundance and diversity in a semi-arid savanna, Southwest Zimbabwe. *Plant Soil.* **383**, 291–299 (2014).

154. P. J. Franco, E. B. Edney, J. F. McBrayer, The distribution and abundance of soil arthropods in the Northern Mojave desert. *J. Arid Environ.* **2**, 137–149 (1979).

155. R. Pellens, I. Garay, Edaphic macroarthropod communities in fast-growing plantations of *Eucalyptus grandis* Hill ex Maid (Myrtaceae) and *Acacia mangium* Wild (Leguminosae) in Brazil. *Eur. J. Soil Biol.* **35**, 77–89 (1999).

156. W. W. Tolbert, V. R. Tolbert, R. E. Ambrose, Distribution, abundance, and biomass of Colorado alpine tundra arthropods. *Arct. Alp. Res.* **9**, 221–234 (1977).

157. G. B. Hewitt, W. H. Burleson, An inventory of arthropods from three rangeland sites in central Montana. *Rangeland Ecology & Management/Journal of Range Management Archives.* **29**, 232–237 (1976).

158. G. C. Loots, P. A. J. Ryke, A comparative, quantitative study of the micro-arthropods in different types of pasture soil. *Zoologica Africana.* **2**, 167–192 (1966).

159. G. B. Hewitt, W. H. Burleson, Arthropods associated with two crested wheatgrass pastures in central Montana. *Rangeland Ecology & Management/Journal of Range Management Archives.* **28**, 301–304 (1975).

160. M. A. Badejo, Seasonal abundance of soil mites (Acarina) in two contrasting environments. *Biotropica.* **22**, 382–390 (1990).

161. M. A. Badejo, N. M. Van Straalen, Seasonal abundance of springtails in two contrasting environments. *Biotropica*. **25**, 222–228 (1993).

162. S. Jing, T. Solhøy, W. Huifu, T. I. Vollan, X. Rumei, Differences in soil arthropod communities along a high altitude gradient at Shergyla Mountain, Tibet, China, *China. Arct. Antarct. Alp. Res.* **37**, 261–266 (2005).

163. M. A. Minor, J. M. Cianciolo, Diversity of soil mites (Acari: Oribatida, Mesostigmata) along a gradient of land use types in New York. *Appl. Soil Ecol.* **35**, 140–153 (2007).

164. S. Salmon, J. Mantel, L. Frizzera, A. Zanella, Changes in humus forms and soil animal communities in two developmental phases of Norway spruce on an acidic substrate. *For. Ecol. Manage.* **237**, 47–56 (2006).

165. D. Wiwatwitaya, H. Takeda, Seasonal changes in soil arthropod abundance in the dry evergreen forest of north-east Thailand, with special reference to collembolan communities. *Ecol. Res.* **20**, 59–70 (2005).

166. E. Haukioja, S. Koponen, "Faunal structure of investigated areas at Kevo, Finland" in *Fennoscandian Tundra Ecosystems: Part 2 Animals and Systems Analysis*, F. E. Wielgolaski, Ed. (Springer, 1975), pp. 19–28.

167. J. A. Wallwork, Distribution patterns and population dynamics of the micro-arthropods of a desert soil in southern California. *J. Anim. Ecol.* **41**, 291–310 (1972).

168. G. Tripathi, S. Ram, B. M. Sharma, G. Singh, Fauna-associated changes in soil biochemical properties beneath isolated trees in a desert pastureland of India and their importance in soil restoration. *Environmentalist* **29**, 318–329 (2009).

169. J. Villarreal-Rosas, J. G. Palacios-Vargas, Y. Maya, Comunidades de microartrópodos relacionadas con costras biológicas de suelo en un matorral desértico en el noroeste de México. *Rev. Mex. Biodivers.* **85**, 513–522 (2014).

170. T. R. Seastedt, D. A. Crossley, Microarthropod response following cable logging and clear-

cutting in the southern appalachians. *Ecology* **62**, 126–135 (1981).

171. M. I. Marques, J. Adis, G. B. dos Santos, L. D. Battirola, Terrestrial arthropods from tree canopies in the Pantanal of Mato Grosso, Brazil. *Rev. Bras. Entomol.* **50**, 257–267 (2006).

172. M. A. Callaham, D. A. Crossley, D. C. Coleman, Soil fauna: Macroarthropods, in *Handbook of Soil Sciences: Properties and Processes*, P. M. Huang, Y. Li, M.P. Sumner, Eds. (CRC Press), pp. 19–26. (2012).

173. M. Lamotte, "The structure and function of a tropical savannah ecosystem" in *Tropical Ecological Systems: Trends in Terrestrial and Aquatic Research*, F. B. Golley, E. Medina, Eds. (Springer Berlin Heidelberg, 1975), pp. 179–222.

174. E. J. Fittkau, H. Klinge, On biomass and trophic structure of the central amazonian rain forest ecosystem. *Biotropica* **5**, 2–14 (1973).

175. A. Legakis, "Community structure and species richness in the Mediterranean-type soil fauna" in *Plant-animal interactions in Mediterranean-type ecosystems*, M. Arianoutsou, R. H. Groves, Eds. (Springer Netherlands, 1994), pp. 37–45.

176. M. Kondoh, H. Watanabe, S. Chiba, A. B. E. Takuya, M. Shiba, S. Saito, Studies on the productivity of soil animals in Pasoh Forest Reserve, West Malaysia: V. Seasonal change in the density and biomass of soil macrofauna: Oligochaeta, Hirudinea and Arthropoda, Mem. *Shiraume Gakuen Coll.* **16**, 1–26 (1980).

177. E. M. Hegazi, M. A. Abd-Elatif, K. S. Moursy, M. F. Maareg, Soil fauna of dry and irrigated farm systems in the Egyptian western desert. *J. Agric. Sci.* **96**, 99–105 (1981).

178. D. E. Reichle, The role of soil invertebrates in nutrient cycling. *Ecol. Bull.* **25**, 145–156 (1977).

179. G. A. de Groot, G. A. J. M. J. op Akkerhuis, W. J. Dimmers, X. Charrier, J. H. Faber, Biomass and diversity of soil mite functional groups respond to extensification of land management, potentially affecting soil ecosystem services. *Front. Environ. Sci. Eng. China.*

4, 15 (2016).

180. S. I. Ghabbour, J. P. C. Da Fonseca, W. Z. A. Mikhail, S. H. Shakir, Differentiation of soil fauna in desert agriculture of the Mariut region. *Biol. Fertil. Soils* **1**, 9–14 (1985).

181. E. Barros, B. Pashanasi, R. Constantino, P. Lavelle, Effects of land-use system on the soil macrofauna in western Brazilian Amazonia. *Biol. Fertil. Soils* **35**, 338–347 (2002).

182. E. Barros, A. Neves, E. Blanchart, E. C. M. Fernandes, E. Wandelli, P. Lavelle, Development of the soil macrofauna community under silvopastoral and agrosilvicultural systems in Amazonia. *Pedobiologia* **47**, 273–280 (2003).

183. E. Barros, M. Grimaldi, M. Sarrazin, A. Chauvel, D. Mitja, T. Desjardins, P. Lavelle, Soil physical degradation and changes in macrofaunal communities in Central Amazon. *Appl. Soil Ecol.* **26**, 157–168 (2004).

184. T. Decaëns, L. Mariani, P. Lavelle, Soil surface macrofaunal communities associated with earthworm casts in grasslands of the Eastern Plains of Colombia. *Appl. Soil Ecol.* **13**, 87–100 (1999).

185. K. J. Hutchinson, K. L. King, The effects of sheep stocking level on invertebrate abundance, biomass and energy utilization in a temperate, Sown Grassland. *J. Appl. Ecol.* **17**, 369–387 (1980).

186. T. G. Wood, R. A. Johnson, S. Bacchus, M. O. Shittu, J. M. Anderson, Abundance and distribution of termites (Isoptera) in a riparian forest in the southern guinea savanna vegetation zone of Nigeria. *Biotropica.* **14**, 25–39 (1982).

187. A. Vasconcellos, Biomass and abundance of termites in three remnant areas of Atlantic Forest in northeastern Brazil. *Rev. Bras. Entomol.* **54**, 455–461 (2010).

188. J. Mathieu, J.-P. Rossi, M. Grimaldi, P. Mora, P. Lavelle, C. Rouland, A multi-scale study of soil macrofauna biodiversity in Amazonian pastures. *Biol. Fertil. Soils* **40**, 300–305 (2004).

189. M. Schaefer, The soil fauna of a beech forest on limestone: Trophic structure and energy budget. *Oecologia* **82**, 128–136 (1990).

190. P. F. Santos, E. DePree, W. G. Whitford, Spatial distribution of litter and microarthropods in a Chihuahuan desert ecosystem. *J. Arid Environ.* **1**, 41–48 (1978).

191. P. Dennis, M. R. Young, I. J. Gordon, Distribution and abundance of small insects and arachnids in relation to structural heterogeneity of grazed, indigenous grasslands. *Ecol. Entomol.* **23**, 253–264 (1998).

192. K. L. King, K. J. Hutchinson, The effects of sheep stocking intensity on the abundance and distribution of mesofauna in pastures. *J. Appl. Ecol.* **13**, 41–55 (1976).

193. H. D. Blocker, "Rangeland invertebrate studies: A review and a look at the future" in *Third midwest prairie conference proceedings*.

194. T. B. Kirchner, The effects of resource enrichment on the diversity of plants and arthropods in a shortgrass prairie. *Ecology* **58**, 1334–1344 (1977).

195. M. Tewari, B. R. Kaushal, Density, diversity and herbivory of aboveground insects in a grassland community of central Himalayan tarai region. *Trop. Ecol.* **48**, 71–78 (2007).

196. B. R. Kaushal, P. C. Joshi, Population, biomass and secondary net production of aboveground insects in a temperate grassland. *Proc. Indian Acad. Sci. Anim. Sci.* **97**, 319–327 (1988).
